# A dual phospholipase system instructs membrane hydrolysis during the final stages of plant autophagy

**DOI:** 10.1038/s41467-026-73116-x

**Published:** 2026-05-14

**Authors:** Julie Castets, Matthieu Buridan, Inés Toboso Moreno, Valérie Wattelet-Boyer, Víctor Sánchez de Medina Hernández, Rodrigo Enrique Gomez, Franziska Dittrich-Domergue, Josselin Lupette, Clément Chambaud, Stéphanie Pascal, Tarhan Ibrahim, Tolga O. Bozkurt, Yasin Dagdas, Frédéric Domergue, Jérôme Joubès, Elena A. Minina, Amélie Bernard

**Affiliations:** 1 University Bordeaux, CNRS, LBM, UMR 5200, Villenave d’Ornon, France; 2 Gregor Mendel Institute (GMI), Austrian Academy of Sciences, Vienna BioCenter (VBC), Vienna, Austria; 3 Vienna BioCenter PhD Program, Doctoral School of the University of Vienna and Medical University of Vienna, Vienna, Austria; 4 Department of Life Sciences, Imperial College London, London, UK; 5 Heidelberg University, Centre for Organismal Studies (COS), Heidelberg, Germany; 6 Department of Molecular Sciences, Uppsala BioCenter, Swedish University of Agricultural Sciences and Linnean Center for Plant Biology, Uppsala, Sweden; 7 Present Address: Aix Marseille Université, CEA, CNRS, BIAM, LGBP, Marseille, France

**Keywords:** Macroautophagy, Proteolysis in plants, Protein trafficking in plants

## Abstract

Autophagy is a conserved intracellular catabolic process, critical for plant stress tolerance. Upon their delivery in the vacuole, how autophagic bodies containing cargo are hydrolyzed to warrant autophagy degradation remains unclear in multicellular organisms. Here, we found that two Arabidopsis phospholipases, LCAT4 and LCAT3, traffic to the vacuolar lumen and converge on autophagic bodies through fundamentally different routes. While LCAT4 directly binds ATG8 and uses autophagy as a transport system to reach the vacuole prepackaged within autophagosomes, LCAT3 traffics to the lytic compartment independently of autophagosome formation. Knocking out both genes causes an accumulation of autophagic bodies accompanied with a reduction in autophagy degradation. In vivo reconstitution demonstrated that LCAT3 can hydrolyse the membrane of autophagic bodies, enabling the activity of LCAT4 to enhance this process. Together, this work sheds light on the vacuolar stages of autophagy, showing that plants have evolved a multi-component pathway for the efficient disruption of autophagosomal membranes as a critical step for the completion of the autophagy pathway.

## Abstract (web summary)

Here the authors show that the Arabidopsis phospholipases LCAT3 and LCAT4 converge on autophagic bodies with differential trafficking pathways and disrupt their membrane in the vacuole thereby guaranteeing autophagy degradation.

## Introduction

Eukaryotes have developed macroautophagy (hereafter referred to as autophagy), an intracellular catabolic pathway which integrates environmental and developmental cues to promote cell homeostasis and adaptation. This process entails the sequestration of intracellular cargo (proteins, protein aggregates, organelles) into specialized vesicles, its trafficking to the lytic compartment of the cell (the lysosomes in mammals and the central vacuole in yeast or plant cells), its degradation therein and the subsequent recycling of the resulting molecules^1^. The importance of autophagy in plant physiology is unequivocal: it is critical for plant survival to virtually all types of stresses and, although plants lacking autophagy can grow in controlled conditions, they show pleiotropic developmental phenotypes including accelerated senescence, defects in seed formation and germination as well as reduced vegetative growth and fecundity^2^. The physiological importance of autophagy is mediated by its two main functions: the degradation of damaged, unwanted or superfluous intracellular material which both detoxifies cells and rewires their activities to meet developmental and environmental demands, and the recycling of autophagy end-products, which reallocates resources when they become scarce and sustains metabolism thereby supporting cell survival^1^. As such, autophagy is defined as a degradation and recycling process.

At present our understanding of autophagy has largely focused on the initial stages of the pathway, *i.e*., the formation of autophagy vesicles, named autophagosomes, and the molecular bases of cargo recognition and sequestration^2–4^. Upon induction, autophagy morphologically starts with the nucleation of a cup-shaped membrane compartment, the phagophore, at which the autophagy machinery sequentially coalesces. This machinery consists in a set of well-characterized proteins named autophagy related proteins (ATG proteins), initially discovered in yeast and largely conserved in mammals and plants. Among those, ATG8 is a central actor of the pathway, which recruitment at the phagophore participates in cargo selection through protein/protein interactions^3,4^. The sequential action of the ATG machinery results in the structurally- and functionally-hyper organized expansion of the phagophore, which progressively grows while recognizing and engulfing autophagy cargo until the fusion of the membranes at the rim of the phagophore which yields a double membrane autophagosome packed with material to be degraded^3–5^. Either directly or upon maturation with late endosomes or multivesicular bodies (MVB), autophagosomes ultimately fuse with the vacuolar membrane^6,7^. At this step, the outer membrane of autophagosomes/amphisomes becomes part of the tonoplast, while their inner membrane containing cargo, are released as a vesicle named autophagic body in the vacuolar lumen^3,8^.

Little is known about the fate of autophagic bodies; yet, their breakdown represents a pivotal entry point into vacuolar degradation as it releases cargo for subsequent hydrolysis and thereby controls the completion of the autophagy pathway and its function as a degradation mechanism. More than a mere passive event, the dismantling of the membrane of autophagic bodies must be tightly controlled in time, to process the large influx of vesicles in the vacuole upon autophagy induction, and in space, to localize membrane disruption at autophagic bodies while preserving the homeostasis of the vacuolar membrane. In particular, the plant lytic vacuole shows distinct features compared to the small and highly mobile lysosomes or to the yeast vacuoles where some aspects of autophagy degradation have been characterized^9,10^. The vacuole represents up to 90% of the whole volume of plant cells^11^; its membrane, the tonoplast, compartmentalizes an acidic sap (pH ~5–5.5)^12^ where hydrolases perform the lytic functions of the cell; consequently, rupture of the tonoplast leads to rapid cell death^13^. Contrarily to yeast or mammals where the membrane of lytic compartments is protected from intralumenal hydrolases by the high glycosylation of resident proteins (forming the so-called glycocalyx^14^), plant vacuolar proteins show little glycosylation^15^ questioning the presence of a protective glycocalyx in plant cells. At this time, how the plant vacuole processes the large influx of autophagic bodies observed upon autophagy induction^1^ and their specific hydrolysis to complete autophagy degradation while maintaining the integrity of its membrane remains completely unknown.

Our proteomic analyses identified an atypical phospholipase A (PLA) called LCAT4 enriched in purified autophagy compartments^16^. Phospholipases A are enzymes which catalyze the hydrolysis of acyl chains from phospholipids, contributing to lipid processing and remodeling, and thereby regulating membrane composition, function, stability and biophysical properties^17^. As such, phospholipases A are involved in numerous developmental and cellular processes including the morphogenesis of endomembrane compartments as well as plant responses to stresses^18^. However, none of these PLA has yet been linked to autophagy in plants and while the biochemical activity of LCAT4 has been partially characterized^19^, its biological function remains unknown. At the starting point of this study, we aimed at addressing the physiological relevance of the association of LCAT4 with autophagy compartments, *i.e*., the potential implication and function of LCAT4 in the autophagy pathway. In this work, combining cell biology, molecular modeling, biochemistry, genetics and in vivo reconstitution, we show that LCAT4 binds ATG8 and uses autophagy to relocalize from the cytosol to the vacuole inside autophagic bodies upon nutrient starvation. We found that the closest homolog of LCAT4, LCAT3, traffics to the vacuole independently of autophagy, where it recognizes the membrane of autophagic bodies. In the absence of both enzymes, autophagic bodies accumulate in the vacuole and autophagic flux is largely reduced. Conversely, expressing a combination of LCAT3 and LCAT4 in yeast cells efficiently disrupts the membrane of autophagic bodies. Together, this study leads us to propose a model in which LCAT3 and LCAT4 act synergistically to support the specific and efficient disruption of autophagic bodies in the vacuole of *Arabidopsis*, thereby unraveling the mechanism and molecular actors involved in the vacuolar stage of autophagy degradation.

## Results

### LCAT4 localizes to autophagy compartments

**Fig. 1:**
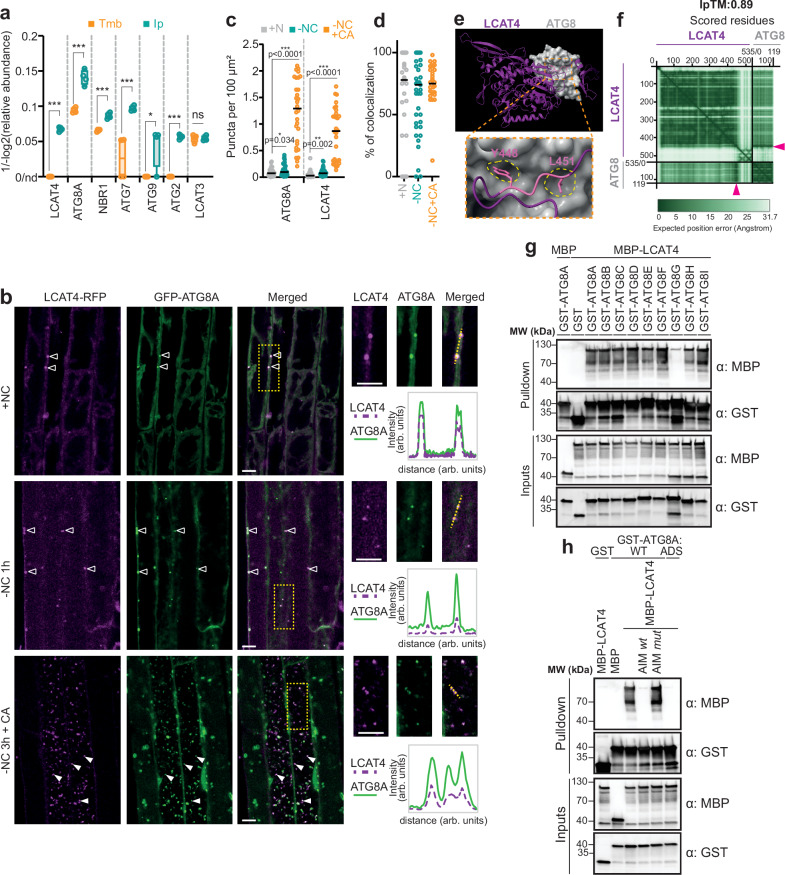
LCAT4 localizes to autophagy compartments and interacts with ATG8A. **a** LCAT4 is enriched in immuno-isolated ATG8A compartments (Ip) compared to the membrane fraction input (Tmb), while LCAT3 is not. Data are presented as box plot, the box extends from the 25th to 75th percentiles, the center line indicates the median, the whiskers show min and max values, all individual values of *n* = 4 biological replicates are plotted. *p* values obtained from two-tailed unpaired *t*-tests are provided in *Source Data*. **b–d** LCAT4 localizes in the cytosol and co-localizes with ATG8A on autophagy compartments. Confocal images of root cells of seedlings co-expressing LCAT4-RFP and GFP-ATG8A placed in +N, -NC 1 h, or in -NC supplemented with concanamycin A (-NC 3 h+CA) (**b**). Empty arrowheads, autophagosomes; full arrowheads, autophagic bodies. Yellow rectangles are enlarged on the right, signal intensity profiles along the dotted lines are plotted below and show co-localization. Scale bar, 10 μm. The experiment was repeated with similar results: +N, *n* = 40 images over 12 roots in 3 independent experiments; -NC, *n* = 39 images/12 roots/3 independent experiments; -NC + CA, *n* = 30 images/9 roots/2 independent experiments. Quantification of (**b**) shows the number of GFP-ATG8A and LCAT4-RFP puncta (**c**) and co-localization between the two proteins (represented as the percentage of RFP puncta colocalizing with GFP puncta, **d**). All individual values are presented with median, *p* values of unpaired two-sided student *t*-test are indicated. AlphaFold3-multimer modeling (**e**) and predicted aligned error (PAE) plot (**f**) place ATG8 in close proximity to the residues Y448 V449 I450 L451 of LCAT4 (see arrow). Y448 and L451 are predicted to bind to the W and L pockets of the ATG8 ADS domain (shown as yellow dashed circles, **e**). LCAT4 binds ATG8 in an AIM dependent manner in vitro. MBP-LCAT4 is pulled-down by each of the nine Arabidopsis ATG8 isoforms (**g**). Interaction is lost with ATG8A^ADS^ or when AIM competition peptides are added (AIM *wt* compared to *mut* peptides, final concentration of 200 µM, **h**). Input and bound proteins were visualized by immunoblotting with anti-GST and anti-MBP antibodies. The experiment was repeated twice with similar results (see Sup. Figure 3a,b).

During autophagy, the formation and subsequent disruption of autophagosomes rely on extensive membrane remodeling events. Lipids and proteins involved in lipid dynamics play critical roles in the instruction of membrane shaping. In that context, and to better understand the molecular bases underlying autophagy, we searched for proteins involved in lipid metabolism in the proteome of autophagy compartments that we pulled down with ATG8A, a core component of the autophagy machinery^16^. Alongside other autophagy-related proteins, we found that a phospholipase A called LCAT4 is enriched in GFP-ATG8A decorated membranes (Fig. 1a), suggesting that this soluble protein localizes in autophagy compartments. To get insights into a possible link between LCAT4 and autophagy, we generated stable *Arabidopsis* lines co-expressing LCAT4-RFP and GFP-ATG8A, to analyze their respective subcellular localization. Using in vivo confocal microscopy of *Arabidopsis* seedlings roots, we observed that under nutrient-rich conditions (+ NC), LCAT4 is mostly found in the cytosol and on puncta co-localizing with ATG8A (Fig. 1b–d). When autophagy is induced, ATG8A is recruited to the phagophore membrane and a portion of the protein remains associated with completed autophagosomes; as such, part of the protein is delivered into the vacuole and found inside autophagic bodies^3^. As expected, when autophagy was induced after 1 h of nutrient deprivation (lack of nitrogen and carbon, -NC), the number of GFP-ATG8A-labeled puncta increased (Fig. 1b, c). Consistently, we observed an increase in the number of LCAT4 puncta (Fig. 1b, c) with a high percentage of colocalization with GFP-ATG8A (Fig. 1d). To observe autophagic bodies, we used concanamycin A (CA) which de-acidifies the vacuole, resulting in a reduction of vacuolar degradation^20^. In this condition, we observed a large accumulation of LCAT4-RFP puncta co-localizing with GFP-ATG8A inside the vacuolar lumen (Fig. 1c, d). Similarly, in lines expressing LCAT4-BFP and YFP-ATG18A, a phagophore marker, BFP signal was found on ring-like structures colocalizing with ATG18A (Supplementary Fig. 1). These results show that LCAT4 is associated with all autophagy compartments, *i.e*., phagophores, autophagosomes and autophagic bodies.

We next explored how LCAT4 was recruited and associated to autophagy compartments. Analyses of the amino acid sequence of LCAT4 found 11 putative ATG8 interaction motifs (AIM; with a consensus site characterized as [W, Y, F][X][X][L, I, V]^21^, see Supplementary Fig. 2a). AlphaFold3-multimer modeling indicated a direct binding between LCAT4 and ATG8 and highlighted residues YVIL^448-451^ in the C-terminal region of LCAT4 as a potential AIM domain associating with the AIM docking site of ATG8 (ADS, Fig. 1e, f; Supplementary Fig. 2b–e). The potential interaction of LCAT4 with ATG8 was thus tested using recombinant proteins and pull-down assays. These analyses showed that MBP-LCAT4 is pulled-down by each of the nine ATG8 isoforms from *Arabidopsis* (ATG8A-I) indicating a direct interaction between ATG8 and LCAT4 (Fig. 1g; Supplementary Fig. 3a). Moreover, this interaction was drastically reduced when a competitive AIM peptide known to bind the ADS domain of ATG8A^22^, was added in the reaction mixture or when we used a form of GST-ATG8A where the ADS domain was mutated (Fig. 1h; Supplementary Fig. 3b). These results show that LCAT4 directly binds to ATG8 and suggest the implication of a canonical AIM-ADS interaction in the localization of LCAT4 to autophagy compartments.

### LCAT4 uses autophagy as a trafficking route to reach the vacuolar lumen upon nutrient starvation

**Fig. 2:**
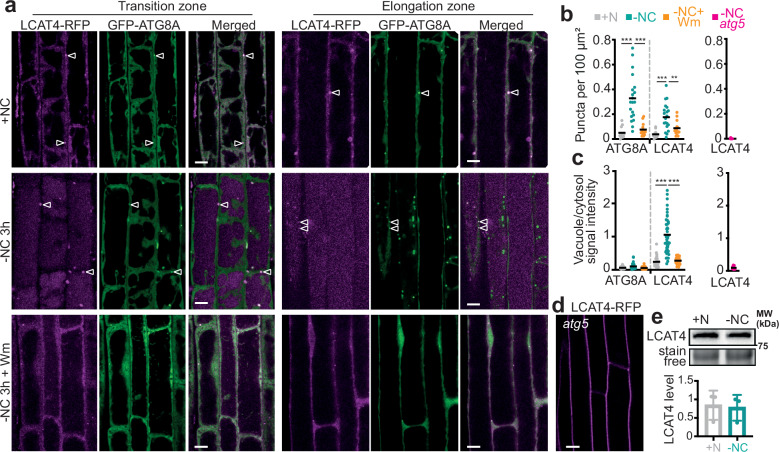
LCAT4 uses autophagy to traffic to the vacuole upon nutrient starvation. **a–d** LCAT4-RFP relocalizes from the cytosol to the vacuolar lumen upon nutrient starvation. Blocking autophagy (by adding Wortmannin or in the *atg5* mutant) restricts LCAT4-RFP in the cytosol. **a** Representative confocal images of seedlings co-expressing GFP-ATG8A and LCAT4-RFP in the transition and elongation zone of the roots. Plants were imaged in +N, -NC 3 h or after -NC 3 h supplemented with Wortmannin (-NC 3 h+Wm). The experiment was repeated three ( + N, -NC+Wm) and four (-NC) independent times with similar results and quantified in (**b**). Arrowheads, autophagosomes; scale bar, 10 µm. **b** Quantification of the number of GFP-ATG8A and LCAT4-RFP puncta of images of (**a**, left panel) and (**d**, right panel). Results are presented as the number of puncta per surface of root area and show all individual values and mean of n number of images: in +N, *n* = 12, in -NC, *n* = 20, in -NC + Wm, *n* = 15, in *atg5*, *n* = 6. **c** Quantification of the LCAT4-RFP signal measured in the vacuole compared to LCAT4-RFP signal measured in the cytosol of images from (**a**). Results are presented as the ratio of vacuole/cytosol signal intensity and show the mean and individual values of: in +N, *n* = 28 cells examined over 8 roots in three independent biological experiments; in -NC, *n* = 43 cells/11 roots/4 independent experiments, in NC+Wm, *n* = 37 cells/ 8 roots/3 independent experiments, in *atg5*, *n *= 14 cells/ 5 roots/1 independent experiment. **d** Confocal image of seedlings of *atg5* mutant expressing LCAT4-RFP after 3 hours in nutrient starvation. The experiment was repeated in 5 independent roots with similar results. Scale bar, 10 μm. **e** The level of LCAT4-RFP is unaffected after 3 h of -NC. Detection of LCAT4-RFP by immunoblot from total proteins extracted after 3 h in liquid +N or -NC. Representative image of *n* = 3 independent experiments (upper panel) showing similar results and quantification of the level of LCAT4 (lower panel), normalized by the loading control; all values are presented with average ± SD. *p* values obtained from two-tailed unpaired *t*-tests are provided in the Source Data file (**b–c**).

Results from Fig. 1 show that LCAT4 is cytosolic in nutrient-rich conditions and localize to autophagosomes and autophagic bodies upon nutrient starvation. Further analyses of the localization of LCAT4 revealed that the cytosolic pool of LCAT4-RFP largely decreases after prolonged period of starvation (-NC 3 h), with a concomitant increase of the protein signal inside the vacuole lumen (Fig. 2a, c; see co-localization with the vacuolar marker BCECF in Supplementary Fig. 3c). Further, immunoblot analyses show that the level of LCAT4 is mostly unaffected in these conditions (Fig. 2e), suggesting that the presence of LCAT4 in the vacuole does not lead, at least initially, to its degradation. Therefore, we concluded that LCAT4 is not a substrate for autophagy degradation but rather transported from the cytosol to the vacuole upon nutrient starvation. Because we found that LCAT4 associates with autophagy compartments in these conditions (Figs. 1, 2), we postulated that the protein uses the autophagy pathway as a trafficking route to reach the vacuole. To test this hypothesis, we analyzed the localization of LCAT4 and ATG8A when autophagy was inhibited either pharmacologically or genetically. Upon Wortmannin (Wm) treatment, an inhibitor of the PI3 kinase complex critical for autophagy^23^, we observed a large reduction of ATG8A puncta compared to untreated cells (Fig. 2a, b), indicating that autophagosome formation is disrupted as expected. In these conditions, the number of LCAT4 puncta also decreases, supporting the genuine association of LCAT4 with autophagosomes (Fig. 2a, b). Further, the LCAT4-RFP signal was found mostly absent from the vacuolar lumen but, rather, restricted to the cytosol (Fig. 2a, c). Similarly, when LCAT4-RFP was expressed in the *atg5* KO line, where autophagosome formation is blocked^24^, we did not observe any LCAT4 puncta, and the signal of the protein was found exclusively in the cytosol (Fig. 2b–d). Together, these results show that LCAT4 relocates from the cytosol to the vacuolar lumen upon nutrient starvation, and that autophagosome formation is required for its transport. Of note, we observed that upon long-term starvation, LCAT4-RFP showed a decrease in protein level that was partially prevented by the addition of concanamycin A (Supplementary Fig. 3d, e), suggesting that LCAT4 is ultimately degraded in the vacuole.

### LCAT4 is dispensable for autophagy during nitrogen and carbon deprivation

**Fig. 3:**
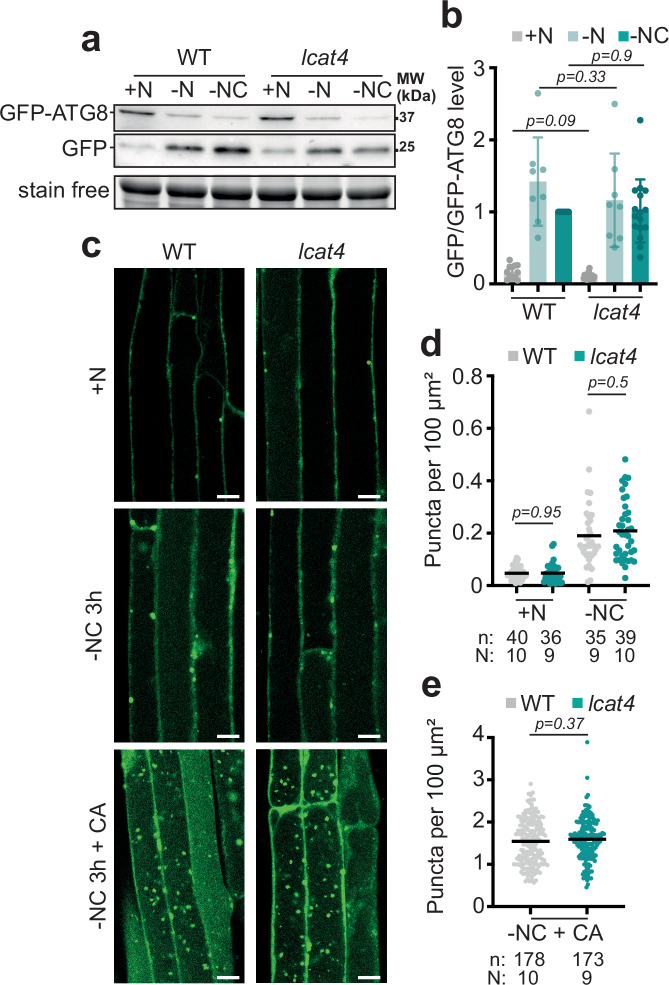
LCAT4 is dispensable for autophagy. **a, b** Autophagy flux is unaffected in the *lcat4* KO mutant compared to WT plants. **a** Detection of GFP-ATG8A degradation and release of free GFP of WT or *lcat4* plants expressing GFP-ATG8A. Seedling were transferred to either rich liquid medium (+ N), liquid medium depleted in nitrogen (-N) or nitrogen and carbon (-NC) during 6 h. Total proteins were extracted and analyzed by immunoblot with anti-GFP antibodies. Stain free images were used as loading control. The experiment was repeated with similar results as indicated and quantified in (**b**). **b** Quantification of the ratio of GFP/GFP-ATG8A relative to that of WT in -NC condition which was set to 1 in each experiment. Results are presented as the average, SD and values of all distinct replicates. In +N and -NC, *n* = 12 (WT), *n* = 16 (*lcat4*); in -N, *n* = 8. **c**,** d** The number of ATG8A-labeled compartments is not affected in the *lcat4* mutant compared to the WT. **c** Representative confocal images of WT or *lcat4* plants expressing GFP-ATG8A. Plants were imaged after 3 hours in +N, -NC, or after 3 h of -NC followed by 2 additional hours of -NC supplemented with concanamycin A (-NC + CA). Scale bar, 10 µm. The experiment was repeated with n independent images over N independent roots with similar results as indicated in the figure and quantified in (**d**). **d** Quantification of GFP-ATG8A puncta in conditions +N and -NC of (**c**). Results are presented as the number of puncta per 100 μm² of root area and show the mean and individual values of n images over N independent plants as indicated in the figure. **e** Quantification of puncta labelled by GFP-ATG8A inside the vacuole in the condition -NC + CA of (**c**). Results are presented as the number of puncta per 100 μm² of root area and show the mean and individual values of n images over N independent plants as indicated in the figure. Statistical differences were assessed using two-tailed unpaired *t*-test (**b**, **d**, **e**).

We next sought to understand the functional relevance of the association of LCAT4 with the autophagy pathway. Although the function of LCAT4 remained unknown in plants, its biochemical activity was partially characterized by expressing the protein in *S. cerevisiae*^19^. In this system, LCAT4 was found to have a phospholipase A_2_ activity, *i.e*., to hydrolyze the acyl chain at the *sn-2* position of a variety of phospholipids. Further, LCAT4 was found to only be active at acidic pH, between 4.5 and 5.5, with an optimal at pH=5.0^19^, which corresponds to the pH of the vacuolar lumen of *Arabidopsis*^12^. Based on our results presented in Figs.1–2, we thus proposed that, mostly residing in the pH-neutral cytosol, LCAT4 is inactive at steady state; upon nutrient starvation, autophagy is induced, transporting LCAT4 into the acidic vacuole where it becomes active. Because of its biochemical activity as a phospholipase, its presence in autophagic bodies and its trafficking inside the vacuole upon autophagy induction, we reasoned that LCAT4 may play a role in the hydrolysis of autophagic bodies in the vacuole during nutrient starvation. To test this hypothesis, we measured the impact of misexpressing *LCAT4* on autophagy, using a T-DNA knocked-out line, *lcat4* (Supplementary Fig. 4a–c) as well as a line in which the expression of *LCAT4* was conditionally knocked-down (*amiRNA:LCAT4*, Supplementary Fig. 4d). First, we measured the rates of autophagic flux using the western-blot-based GFP-ATG8 processing assay. GFP-ATG8 is associated with autophagosomal membrane and a portion of the protein is delivered in the vacuole inside autophagic bodies. Upon the disruption of the membrane of autophagic bodies, GFP-ATG8 is delivered in the vacuolar lumen where it is rapidly degraded, while the more stable GFP moiety accumulates^25^. Therefore, measuring the ratio between the level of GFP-ATG8A and that of its degradation product, free GFP, quantitatively reflects the rate of autophagic degradation. To test whether LCAT4 was required for autophagy activity, seedlings of *LCAT4* KO and KD lines were placed in nutrient rich liquid medium (+ N) or nutrient-depleted liquid medium (lacking either nitrogen, -N or a combination of nitrogen and carbon, -NC) and compared to WT plants. Upon -N or -NC, autophagy was induced in WT plants, as seen by the reduction of the GFP-ATG8A signal and the concomitant increase in free GFP (Fig. 3a, b). The ratio of GFP/GFP-ATG8A was not significantly different than that of WT in plants KO or KD for *LCAT4* (Fig. 3a, b and Supplementary Fig. 4f, g, respectively) indicating that autophagy flux is not affected by the absence of LCAT4. Similarly, in vivo quantitative analyses of the number of autophagosomes (Fig. 3c, d) and autophagic bodies (Fig. 3c, e) labelled by GFP-ATG8A in the roots of 7-day-old seedlings showed no significant differences between WT and *lcat4* plants. Together, these results show that the absence of LCAT4 does not significantly impact autophagosome formation, autophagic body accumulation or autophagic degradation.

### LCAT3 is a potential redundant phospholipase for autophagy

The absence of a significant phenotype in the *lcat4* mutant prompted us to address the potential redundancy of additional phospholipases in the process of autophagic body degradation. LCAT4 belongs to a small multigenic family comprising four members: LCAT1, LCAT2 and LCAT3 (Supplementary Fig. 5a). LCAT3 shares 53% identity with LCAT4 (Supplementary Fig. 5a, 6), was detected in the vacuolar sap by proteomic analyses^26^ and was previously characterized as an acidic phospholipase A_1_ by heterologous expression in yeast^27^. LCAT1 shares 23% identity with LCAT4 (Supplementary Fig. 5a), is a transmembrane protein previously found in a vacuolar proteome^28^ and is predicted to have a hydrolase activity although it remains uncharacterized at this time. In contrast, LCAT2, also known as PSAT1, shares 20% identity with LCAT4 (Supplementary Fig. 5a) and has been previously demonstrated to have an acyltransferase activity involved in the formation of steryl esters^29^. Based on this data, we postulated that LCAT2 was unlikely to have a redundant function with LCAT4 and focused our research on LCAT3 and LCAT1.

**Fig. 4:**
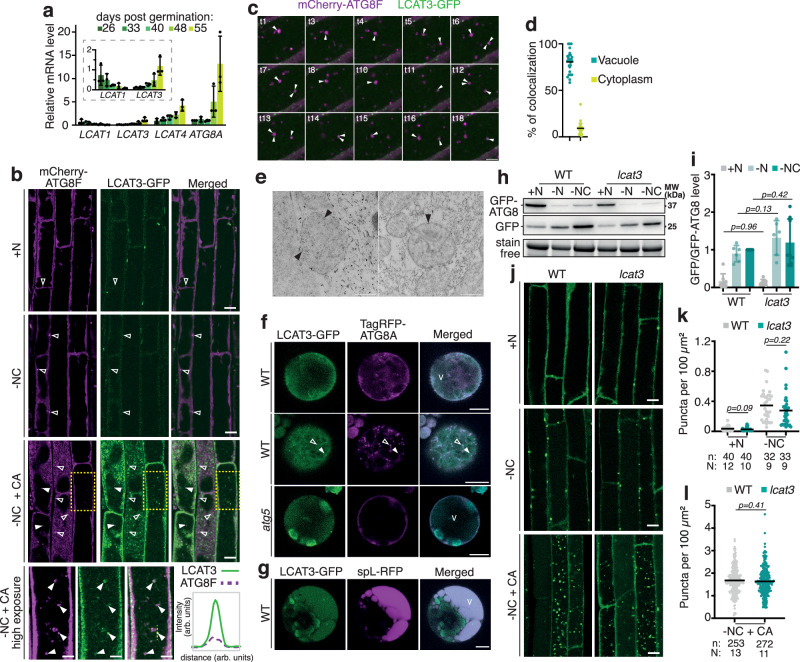
LCAT3 localizes on autophagic bodies yet knocking out *LCAT3* has no effect on autophagy activity. **a** Abundance of transcripts in leaves, relative to *ATG8A* at day 26. Results show average ± SD and values of *n* = 3 biological replicates. LCAT3 localizes on autophagic bodies (full arrowheads) but not autophagosomes (empty arrowheads) in confocal images of roots of plants co-expressing LCAT3-GFP and mCherry-ATG8F after 3 h of +N, -NC or -NC + CA (**b**, see increased signal intensity of LCAT3-GFP in -NC + CA; top 3 row, scale is 10 µm; yellow rectangles are enlarged on the lowest panel, scale is 5 µm. Signal intensity along the dotted line is plotted on the right) or time-lapse images in -NC + CA (**c**, time in seconds indicated in upper left corner; scale is 5 µm). Repeated with similar results as indicated and quantified in (**d**) showing percentage of LCAT3 puncta colocalizing with ATG8F in the vacuole (*n* = 26 images/12 roots/5 experiments) or in the cytoplasm (*n* = 25 images/11 roots/4 experiments). **e** Representative images of immunolabeling and EM imaging showing LCAT3-GFP (arrows) on the membrane of autophagic bodies. 174 autophagic bodies were imaged, 57 of which showed gold particles. Scale, 0.5 µm. Confocal images of protoplasts co-expressing LCAT3-GFP and TagRFP-ATG8A show LCAT3 in the vacuole (V) in either WT (top row in **f** and co-localization with the vacuolar lumen marker sPL-RFP in **g**) or *atg5* protoplasts (bottom row in **f**). Middle row shows cortical cytoplasm containing autophagosomes (arrowheads) and LCAT3 puncta (filled arrowheads). Scale, 10 µm. Representative of two independent experiments with similar results. Autophagy flux (**h**,** i**) and confocal analyses of GFP-ATG8A puncta (**j**, scale bar, 10 μm; **k**,** l**) were performed and quantified as in Fig. 3 in WT or *lcat3*. Experiments repeated with similar results, quantified and presented as: (**i**) the average of GFP/GFP-ATG8A ± SD with values of independent experiments: +N and -NC, *n* = 8; -N, *n* = 6; (**k**,** l**) the mean number of ATG8A puncta per root area with individual values of *n* images over N independent plants as indicated in the figure, in the cytoplasm (**k**) or in the vacuole (**l**). Statistical differences were assessed using two-tailed unpaired *t*-test.

Under stress-inducing conditions or during plant senescence, an increase in the expression of autophagy-related genes support autophagic induction and activity, notably improving stress tolerance^30^. To investigate whether LCAT3 and LCAT1 can participate in the autophagy pathway, we first analyzed their expression profiles in response to autophagy induction. First, the relative abundance of *LCAT1* and *LCAT3* transcripts was measured and compared to that of *LCAT4* and *ATG8A* during leaf development using RT-qPCR. Similar to *ATG8A*, *LCAT3* and *LCAT4* were found up-regulated during plant ageing; conversely, the transcripts of *LCAT1* decreased under the same conditions (Fig. 4a). In addition, we measured the expression level of *LCATs* genes in 7-day-old seedlings after 6 hours of nitrogen and carbon starvation. Similar to what we measured during senescence, we recorded an increase in the transcripts of *LCAT4* and *LCAT3*, and decreased abundance of *LCAT1* in these conditions (Supplementary Fig. 5b). The increased expression of *LCAT3*, alongside *LCAT4*, in conditions where autophagy is induced, either upon nutrient deficiency or senescence, supports its potential involvement in the autophagy pathway which was experimentally tested (see below). Although we acknowledge that gene expression does not necessarily predict function, the repression of *LCAT1* led us to not consider this gene further in this study, leaving future investigations to explore whether it has a role in autophagy.

To test whether LCAT3 participates in the autophagy pathway, we analyzed the subcellular distribution of the protein in regard to autophagy compartments by generating stable *Arabidopsis* lines co-expressing LCAT3-GFP and mCherry-ATG8F. Imaging the roots of 7-day-old seedlings, under rich or -NC conditions using confocal microscopy, we observed that the LCAT3-GFP signal was very weak, even when the transgene was expressed under the control of the constitutive pUBQ10 promoter (Fig. 4b). In these conditions, LCAT3-GFP was mostly found in the cytosol or on cytosolic puncta that do not co-localize with mCherry-ATG8F. This result indicates that LCAT3 is not present on autophagosomes, in contrast to LCAT4 which was found to be recruited to early autophagy compartments (Fig. 1, Supplementary Fig. 1). This difference in localization between the two proteins may be specified by their diverging C-terminal parts: although they show high sequence identities overall, LCAT3 is shorter and lacks the C-terminal tail of LCAT4 where the predicted ATG8 interacting domain is located (Supplementary Fig. 6a, b). Accordingly, AlphaFold3 models did not predict an interaction between LCAT3 and ATG8 (Supplementary Fig. 6a–d) which is consistent with results in Fig. 1a where LCAT3, in contrast to LCAT4, was not found enriched in ATG8-immunoisolated compartments.

In contrast to -NC conditions, where LCAT3 did not co-localize with ATG8, when plants were additionally treated with concanamycin A (which enables the visualization of GFP signal in the vacuole, otherwise quenched by the acidity of its lumen) we observed LCAT3 inside the vacuole, on bright foci associated with ATG8-labeled compartments (Fig. 4b–d). This result indicates that LCAT3 traffics to the vacuolar lumen and localizes on autophagic bodies. This was confirmed by immunogold labelling and transmission electron microscopy analyses of LCAT3-GFP expressing plants showing gold particles on the membrane of autophagic bodies (Fig. 4e). The absence of LCAT3 on autophagosomes coupled with the presence of LCAT3 in the vacuole suggests that, in contrast to LCAT4, LCAT3 traffics to the vacuole independently of autophagy. To test this hypothesis, we transiently expressed LCAT3-GFP in cotyledons of the *atg5* mutant, impaired in autophagosome formation, in comparison to that of WT plants. In these experiments, performed in autophagy inducing conditions and in the presence of concanamycin A, LCAT3-GFP was observed in the vacuole of WT cotyledons, either as a diffuse signal or on puncta that co-localize with mCherry-ATG8F-marked autophagic bodies (Supplementary Fig. 7c, d). In the same conditions, LCAT3-GFP was also observed in vacuoles of *atg5* cotyledons but strictly as a diffuse signal (Supplementary Fig. 7c) implying that autophagosome formation is dispensable for the transport of LCAT3 in the vacuole. These results were further recapitulated by expressing LCAT3-GFP in *Arabidopsis* protoplasts showing LCAT3-GFP in the vacuolar lumen in both WT protoplasts (Fig. 4f, g) and in *atg5* protoplasts (Fig. 4f). Consistent with data from Fig. 4b, LCAT3 was also observed on cytosolic puncta that differ from ATG8-labeled autophagosomes (Fig. 4f, middle panel). Despite LCAT3 trafficking route remaining to be characterized, our results show that a portion of LCAT3 localizes in the vacuole lumen, independently of autophagy, and can recognize and associate with autophagic bodies.

### LCAT4 and LCAT3 control autophagy degradation in the vacuole

The homology between LCAT4 and LCAT3, as well as their localization on autophagic bodies, suggest that these two phospholipases might have redundant or synergistic roles in autophagy. To test this hypothesis, we collected a *lcat3* T-DNA KO mutant, generated a conditional *LCAT3* KD mutant and constructed a double *lcat3 lcat4* KO mutant by crossing *lcat3* and *lcat4* T-DNA insertion lines (Supplementary Fig. 4a–c,e). To measure autophagy, the GFP-ATG8A construct was introgressed in the aforementioned lines which were used to perform the GFP-ATG8 degradation assay and to quantify the number of autophagy compartments in vivo. In the *lcat3* KO line, we did not observe significant defects in autophagic flux (Fig. 4h, i) or in the number of GFP-ATG8A-labeled autophagosomes or autophagic bodies (Fig. 4j–l). In contrast, when the expression of *LCAT3* was reduced for a few hours prior to autophagy induction, in the conditional *amiRNA:LCAT3* line, we detected a slight decrease in autophagic flux in -N conditions (Supplementary Fig. 7a, b). These results suggest that LCAT3 has a function in the autophagy pathway and that its complete absence in the *lcat3* KO line is likely complemented by a redundant activity.

**Fig. 5:**
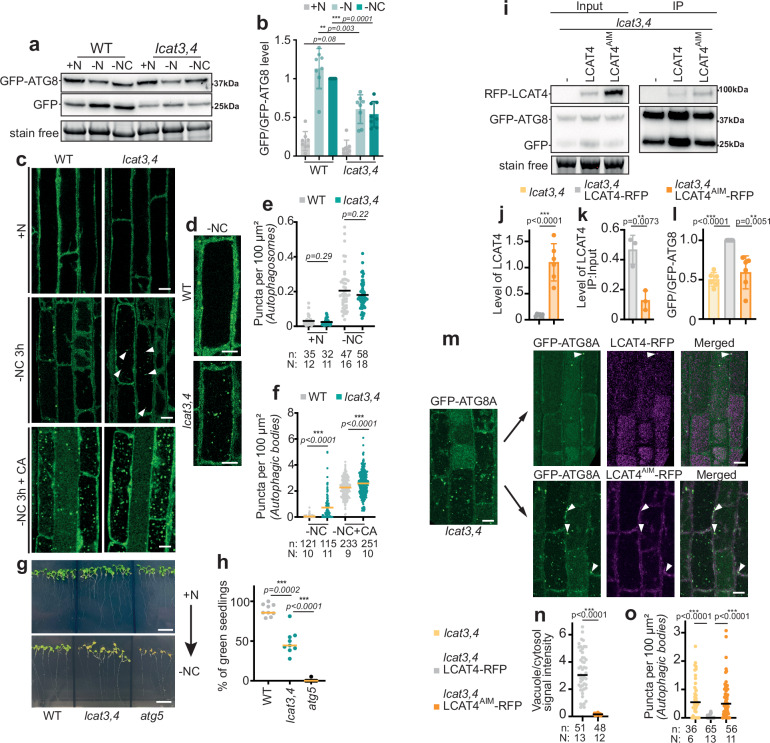
Knocking out LCAT4 and LCAT3 causes defects in autophagy activity and accumulation of autophagic bodies. GFP-ATG8A processing assay was performed as in Fig. 3a (**a**) in *lcat3,4* and WT and quantified (**b**) showing average GFP/GFP-ATG8A ± SD with *n* = 8 independent experiments. **c–f** Confocal analyses of GFP-ATG8A (**c**,** d**, scale, 10 μm) were performed as in Fig. 3c showing autophagic bodies in the vacuole of *lcat3,4* compared to WT in -NC conditions (see arrowheads in **c** and representative cells in **d**). The experiment was repeated with similar results and quantified showing mean numbers of ATG8A puncta per root area with individual values of *n* images over N independent plants as indicated, in the cytoplasm (**e**) or vacuole (**f**, autophagic bodies). *lcat3,4* is less tolerant to prolonged nutrient starvation than WT. Representative images (**g**) quantified in (**h**) as percentage of mostly green seedlings compared to total seedlings and presented as the average and individual values of *n* = 9 replicates. Scale, 1 cm. Expressing LCAT4-RFP in *lcat3,4* restores autophagy activity while LCAT4^AIM^-RFP does not. Images of immunoblot analyses of total protein lysates (input, 10%) and pulldowns with GFP-ATG8A (IP) (**i**) are representative of repeated experiments quantified in (**j–l**, average ± SD), showing the accumulation of LCAT4^AIM^-RFP in total lysates (**j**, *n* = 6) and decrease in ATG8 interaction compared to LCAT4-RFP (**k**, relative to input amount of each protein, *n* = 3). GFP-ATG8 processing assay shows that expressing LCAT4-RFP increases autophagy degradation compared to *lcat3,4* or LCAT4^AIM^-RFP (**l**, *n* = 6, GFP/GFP-ATG8 ratio relative to that of LCAT4-RFP expressing plants which was set to 1). **m–o** Confocal analyses of plants treated with AZD8055 (2 h) showing GFP-ATG8A labelled autophagosomes (arrows) or autophagic bodies, and LCAT4-RFP localization in *lcat3,4* complemented with either LCAT4 or LCAT4^AIM^. Scale, 10 μm. Images are representative of n cells over N plants as indicated in (**m**) and quantified in (**n**, LCAT4 vacuole/cytosol signal intensity) and (**o**, number of autophagic bodies) showing all n individual values with median. Statistical differences were assessed using two-tailed Mann-Whitney test (**b**, **h**), two-tailed unpaired *t*-test (**e**, **f**, **j**, **k**, **n**,** o**) or two-tailed one-sample *t*-test (**l**).

In contrast to single *lcat3* or *lcat4* mutants, analyses of the autophagy flux using the GFP-ATG8 processing assay in the double *lcat3 lcat4* KO line (hereafter *lcat3,4*), showed a decrease in the GFP/GFP-ATG8 ratio, approximately 50% lower than in WT, under either nitrogen- or combined nitrogen and carbon-depleted conditions (Fig. 5a, b). These results show that knocking out both *LCAT3* and *LCAT4* slows down autophagy activity and suggest that LCAT3 and LCAT4 have redundant and/or synergistic effects in the autophagy pathway. To determine the stage of the pathway at which LCAT3 and LCAT4 participate during autophagy, we analyzed the number and distribution of autophagic structures in the *lcat3*,*4* double mutant. We assessed autophagosome formation, by counting the number of ATG8A puncta in the cytosol in rich conditions (+ NC) or in autophagy-inducing conditions (-NC, 3 h). As seen in (Fig. 5c, e), these analyses showed no significant differences in the *lcat3,4* double mutant compared to WT plants. This shows that the autophagy defects observed in the absence of LCAT3 and LCAT4 are not caused by a reduction in autophagosome formation or by an inhibition of autophagosome to vacuole trafficking. Strikingly, these analyses revealed a major difference between *lcat3,4* and WT plants in the vacuole: in -NC conditions, we observed an accumulation of GFP-ATG8A puncta inside the vacuolar lumen of the *lcat3,4* double mutant which are barely ever observed in cells of WT plants (see -NC conditions in Fig. 5c, d, f). This phenotype was recapitulated in an alternative autophagy-inducing condition, *i.e*., upon treatment with the TOR inhibitor AZD-8055 (AZD, Supplementary Fig. 8) demonstrating the robustness of this result. Additionally, when concanamycin A was further added after 3 hours of prior autophagy induction, the number of GFP-ATG8A puncta in the vacuole increased in both genotypes with a significant greater abundance in the double *lcat3,4* mutant compared to WT (see -NC + CA conditions in Fig. 5c, f and similar phenotype upon AZD + CA treatment in Supplementary Fig. 8). These results, in contrast to the deletion of either *LCAT4* or *LCAT3* which showed no impact on autophagy compartments (Fig. 3c, e and Fig. 4j, l, respectively), reveal that the *lcat3,4* mutant accumulates autophagic bodies within the vacuole, indicating that their proper degradation requires LCAT3 and LCAT4 and suggesting partial redundant activities of the proteins. Further, the concomitant accumulation of autophagic bodies and reduction of autophagy flux in the *lcat3,4* mutant demonstrate the importance of autophagic body disruption for the completion of autophagy. Nevertheless, the 50% remaining autophagy activity detected in the *lcat3,4* mutant (Fig. 5a, b) suggests that additional proteins, yet to be identified, are involved in this process. In fact, compared to WT plants, the *lcat3,4* mutant showed similar germination, root length, vegetative growth or onset of senescence, unlike *atg* mutants where autophagy is completely abolished (Supplementary Fig. 9). These results show that the absence of the two proteins, and the corresponding 50% reduction in autophagy flux has no major effect on plant development in rich conditions. In contrast, we observed an intermediate phenotype between that of *atg5* and that of WT when *lcat3,4* seedlings were placed in -NC for 10 days (Fig. 5g, h). While none of the *atg5* mutants were able to cope with prolonged starvation, most WT seedlings were found to be mostly green when the *lcat3,4* mutant displayed a mild yet significant senescing phenotype. Together, these analyses show that LCAT3 and LCAT4 are components of the autophagy machinery, and participate, or control, the hydrolysis of autophagic bodies at the antepenultimate step of the pathway.

Based on our results of Figs. 1, 2 showing that LCAT4 interacts with ATG8 in vitro and that LCAT4 is observed within the vacuolar lumen upon autophagy induction, we proposed that the ATG8-LCAT4 interaction controls the trafficking of LCAT4 to the vacuole. To test this hypothesis and assess the relevance of the predicted AIM domain of LCAT4 we complemented the *lcat3,4* mutant with either LCAT4-RFP or LCAT4^AIM^-RFP (in which the residues YVIL448-451 were mutated to alanines). Co-immunoprecipitation (CoIP) experiments presented in Fig. 5i–l show that LCAT4-RFP co-immunoprecipitates with GFP-ATG8A confirming in vivo interaction between the two proteins. While LCAT4^AIM^-RFP was found largely accumulating in total protein lysates compared to LCAT4-RFP (Fig. 5j), the amount of protein pulled-down by GFP-ATG8 decreased by about 70% showing that the AIM domain of LCAT4 is an important element for its interaction with ATG8 (Fig. 5k). To test the impact of a reduction in LCAT4-ATG8 interaction on LCAT4 localization, we performed confocal imaging analyses of *lcat3,4* plants expressing LCAT4^AIM^-RFP compared to LCAT4-RFP. These analyses recapitulated our observations from Fig. 2, showing that upon autophagy induction, LCAT4-RFP is found on occasional puncta co-localizing with GFP-ATG8A in the cytosol and mostly accumulate within the vacuole (Fig. 5m, upper panel, quantified in **n**). In contrast, LCAT4^AIM^-RFP was found largely restricted in the cytosol where it showed a diffuse signal (i.e., did not form puncta, Fig. 5m, lower panel, quantified in **n**) and did not co-localize with autophagosomes. A faint LCAT4^AIM^-RFP signal was also observed within autophagic bodies, either due to the residual ATG8 interaction or to passive intake of cytosolic material. To test the relevance of LCAT4-ATG8 interaction and LCAT4 localization for its function in autophagy we measured autophagy degradation in *lcat3,4* compared to *lcat3,4* complemented with either LCAT4-RFP or LCAT4^AIM^-RFP. GFP-ATG8 processing assays show that expressing LCAT4-RFP increases autophagy flux compared to *lcat3,4* while LCAT4^AIM^-RFP does not (Fig. 5i, l). Consistently, expressing LCAT4-RFP abolished the accumulation of autophagic bodies observed in *lcat3,4* while LCAT4^AIM^-RFP showed an autophagic body density similar to that of *lcat3,4* (Fig. 5m, o). Together, these results show that LCAT4 interacts with ATG8A in vivo in an AIM-dependent manner and indicate that this interaction controls the recruitment of LCAT4 to autophagosomes and its vacuolar trafficking. A reduction in LCAT4-ATG8 interaction results in an accumulation of the protein in the cytosol, a failure to degrade autophagic bodies and to restore autophagy flux, further supporting the crucial function of LCAT4 and its trafficking for the autophagy pathway.

### LCAT3 and LCAT4 act synergistically to disrupt the membrane of autophagic bodies

**Fig. 6:**
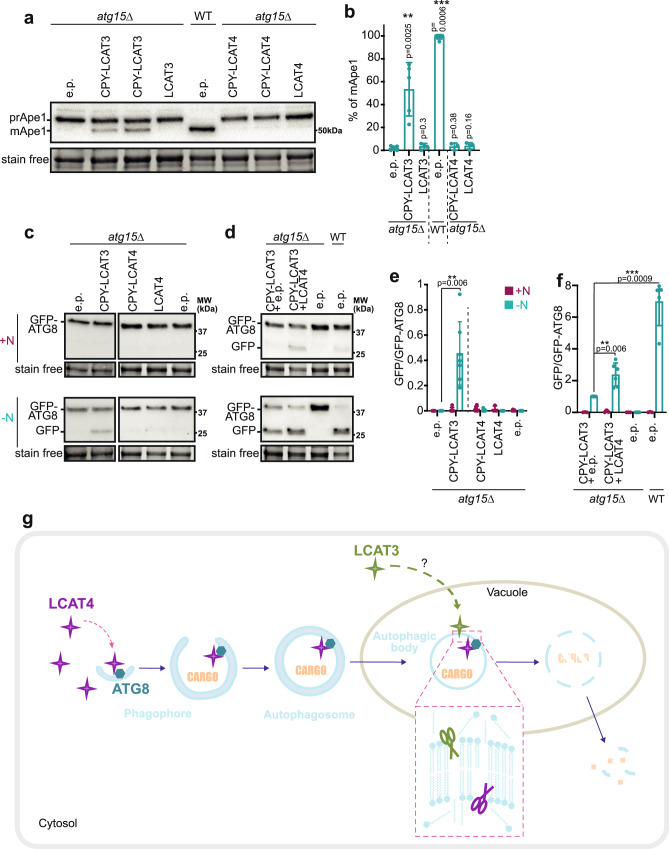
LCAT3 and LCAT4 can disrupt the membrane of autophagic bodies. LCAT3 partially complements Ape1 processing in *atg15Δ*. Immunoblot analyses (**a**) of Ape1 in WT or *atg15Δ* cells transformed with empty plasmid (e.p.), CPY-LCAT3 (2 replicates), LCAT3, CPY-LCAT4 (2 replicates) or LCAT4. The experiment was repeated with similar results and quantified (**b**) showing the average percentage of mApe1 (over total Ape1) ± SD with values of independent replicates: *n* = 7, *n* = 5, *n* = 4, *n *= 7, *n* = 3, *n* = 4 (left to right). Expressing CPY-LCAT3 partially restores autophagy degradation in *atg15Δ* cells (**c**, **e**) and autophagy flux is further enhanced when CPY-LCAT3 and LCAT4 are co-expressed (**d**, **f**). Immunoblot analyses of GFP-AtATG8A processing assays in *atg15Δ* transformed with empty plasmid (e.p.), CPY-LCAT3, CPY-LCAT4 or LCAT4 (**c**) or co-transformed with CPY-LCAT3 + e.p or CPY-LCAT3 + LCAT4 compared to WT cells transformed with the corresponding empty plasmids (**d**). Yeast cells were grown in rich selective media until mid-log phase (+ N) prior to transfer to nutrient starvation (-N). Stain free images were used as loading control. Experiments from (**c**) were repeated and quantified in (**e**) with results showing average GFP/GFP-ATG8 ± SD with values of independent replicates: *n* = 4, 4, 7, 7, 3, 4, 6, 6, 5, 6 (left to right). Experiments from (**d**) were repeated and quantified in (**f**) with results showing average GFP/GFP-ATG8 (relative to that of CPY-LCAT3 + e.p. which was set to 1)  ± SD with values of independent replicates: *n *= 3, 6, 3, 6, 3, 5, 3, 5 (left to right). Statistical differences were assessed using two-tailed Mann-Whitney tests (**b**, **e**) and two-tailed one sample *t*.test (**f**), significant *p* values are indicated. **g** Working model of autophagic bodies hydrolysis by LCAT3 and LCAT4. LCAT4 is recruited from the cytosol to the phagophore by interacting with ATG8, thereby trafficking inside autophagosomes and autophagic bodies to the vacuolar lumen. LCAT3 traffics to the vacuolar lumen independently of autophagy via a yet to be characterized pathway. LCAT3 and LCAT4 converge on autophagic bodies where they both hydrolyse the lipids composing their membrane, releasing cargo for degradation and subsequent recycling.

Once the autophagic bodies are delivered inside the vacuolar lumen, the disruption of their membrane is a *sine qua non* condition for autophagy progression: it enables the release of the cargo for subsequent degradation and recycling, thereby completing autophagy activity. Further, this process must be tightly regulated by specific lipid-hydrolytic enzymes, able to specifically recognize and target the membrane of autophagic bodies. Based on the localization of LCAT3 and LCAT4 (Figs.4, 1), on the nature of their enzymatic activity as phospholipases^19,27^ and on their impact on the autophagy pathway (Fig. 5) we hypothesized that they participate in the hydrolysis of the membrane of autophagic bodies. To test this potential function, we set up a system in which LCAT3 and LCAT4 were expressed in yeast cells that are genetically impaired for the degradation of autophagic bodies. In the budding yeast *S. cerevisiae*, a single protein called Atg15, a phospholipase B, is required for the breakdown of autophagic bodies^9,10^. Although there is no sequence homologue for Atg15 in plants^31^, we proposed to challenge our idea that LCAT3 and LCAT4 could be functional analogues of this phospholipase in autophagy by testing whether their expression can complement the absence of Atg15 in yeast. To monitor the disruption of autophagic bodies in LCAT3- or LCAT4-expressing *atg15Δ* cells, we first followed the level of the mature form of the vacuolar-resident aminopeptidase 1, Ape1. Ape1 uses a selective type of autophagy, called the Cvt pathway (Cytoplasm-to-Vacuole Targeting), to traffic to the vacuole; inside the vacuolar lumen the prApe1 propeptide is cleaved by vacuolar proteases thus converting Ape1 into a mature and active form (mApe1). In the absence of Atg15, prApe1 reaches the vacuolar lumen but remains entrapped inside autophagic bodies, preventing its maturation^9^ (Fig. 6a, b, compare the empty plasmid condition (e.p.) to WT cells). Expressing either *LCAT3* or *LCAT4* in *atg15Δ* cells did not result in the formation of mApe1, indicating that, at steady state, the proteins cannot rescue the *atg15Δ* phenotype, *i.e*., cannot disrupt autophagic bodies (Fig. 6a, b). In contrast, when LCAT3 was fused to the signal sequence of carboxypeptidase Y (CPY) which addresses protein inside the vacuolar lumen^9^ (Supplementary Fig. 10a, b), we detected mApe1 indicating that a portion of autophagic bodies were hydrolyzed (Fig. 6a, b). Previous work on LCAT3 identified its catalytic triad, consisting of S177, D384, and H409 and showed that replacing S177 to an alanine prevented the phospholipase activity of the protein^27^. Compared to CPY-LCAT3^WT^, *atg15Δ* cells expressing CPY-LCAT3^S177A^ failed to accumulate mApe1 indicating that the phospholipase activity of LCAT3 is required for its capacity to breakdown autophagic bodies (Supplementary Fig. 10c). From these results we concluded that, when expressed in the vacuole, LCAT3 can recognize and hydrolyze the membrane of autophagic bodies in yeast. In contrast, when expressing LCAT4, even when it was fused to the CPY vacuolar-addressing peptide, we did not detect mApe1 (Fig. 6a, b), showing that LCAT4 alone cannot complement the *atg15Δ* phenotype and disrupt the membrane of autophagic bodies.

From our results in Figs. 1–2, 5, we propose that LCAT4 is recruited to the phagophore membrane by its interaction with ATG8 and traffics inside the autophagosomes/autophagic bodies to reach the vacuolar lumen. In contrast, we found that LCAT3 traffics to the vacuole independently of autophagy (Fig. 4b, c, f, g; Supplementary Fig. 7c, d) thus suggesting that it may recognize the outer leaflet of the membrane of autophagic bodies either in *Arabidopsis* (Fig. 4b, c) or in yeast cells (Fig. 6a, b). Based on this data, we postulated that LCAT3 and LCAT4 act synergistically, with LCAT3 starting from the outer leaflet, providing initial membrane disruption to activate LCAT4 localized in the inner leaflet of the vesicular membrane. To test this hypothesis, we swapped the endogenous Atg8 from *S. cerevisiae* to GFP-ATG8A from *Arabidopsis* at the Atg8 locus and tested the impact of iterative expression of CPY-LCAT3 and LCAT4 on GFP-ATG8A processing, as a proxy for autophagic body disruption and autophagy degradation. Our results recapitulated the observation from the analyses of Ape1 in Fig.6a, b by showing that CPY-LCAT3 promotes the degradation of GFP-ATG8A resulting in the accumulation of free GFP upon autophagy induction by nitrogen starvation (Fig. 6c, e; left panel) while expressing either LCAT4 or CPY-LCAT4 did not result in GFP-ATG8A processing (Fig. 6c, e; right panel). However, when we mimicked plant trafficking/localization scenarios, by co-expressing GFP-ATG8A and LCAT4 (in the cytosol) together with CPY-LCAT3 (in the vacuole) in *atg15Δ* cells, we observed a significant increase in autophagy activity compared to the expression of CPY-LCAT3 alone (Fig. 6d, f). In contrast, co-expressing LCAT4^S182A^ (where the residue S182 of its proposed catalytic site was replaced by an alanine) with CPY-LCAT3 did not lead to an increase in autophagy activity compared to CPY-LCAT3 alone (Supplementary Fig. 10d) suggesting that the phospholipase activity of LCAT4 is relevant for its function in autophagic body turn-over. Together, these results indicate that both LCAT3 and LCAT4 can hydrolyze the membrane of autophagic bodies in yeast cells. It further supports the hypothesis that the activity of LCAT4 requires that of LCAT3 and suggests that both enzyme act synergistically to instruct the disruption of autophagic bodies as a prerequisite for the completion of autophagy degradation.

## Discussion

The degradation and recycling of proteins, organelles and pathogens through the autophagy pathway play critical roles in plant development and plant tolerance to environmental stresses^2^. Yet, while our understanding of the formation, cargo packing and trafficking of autophagy vesicles has greatly increased^3^, what occurs inside the vacuole during the actual degradation and recycling steps of the autophagy pathway remain uncharacterized in plants and poorly described across models. Here we uncover the role of two phospholipases, LCAT3 and LCAT4, in the disruption of autophagic bodies in the vacuole (Figs. 5, 6). We show that preventing this process leads to a large decrease in autophagy flux (Fig. 5) supporting the critical relevance of this membrane remodeling event in the completion of the autophagy pathway. Therefore, we propose that both LCAT3 and LCAT4 are part of the machinery of autophagy. From our results we further draw a model according to which plants have evolved a multi-component mechanism to efficiently instruct the disruption of autophagic bodies (Fig. 6g). LCAT4 is recruited to the phagophore membrane through a direct interaction with ATG8, and remains associated with autophagosomes with which it traffics to the acidic vacuolar lumen (Figs. 1, 2). Our data, and that of others, found LCAT3 within the vacuolar lumen and we show that it co-localizes with autophagic bodies (Fig. 4b–d, Supplementary Fig. 7c, d, and identification of LCAT3 in the proteome of the vacuolar sap in ref.^31^). However, in contrast to LCAT4, autophagy is dispensable for its transport to the vacuole (Fig. 4g, h, Supplementary Fig. 7c, d). Therefore, we propose that LCAT3 may recognize the outer leaflet of autophagic bodies while LCAT4 is found inside these compartments, although this remains to be directly demonstrated. Unlike single *lcat3* or *lcat4* mutants which show little to no autophagy defects, the double *lcat3,4* knock out shows a large reduction in autophagy flux and a concomitant accumulation of autophagic bodies in the vacuole (Fig. 5). We thus propose that a synergy between LCAT3 and LCAT4 is required to efficiently promote the degradation of autophagic vesicles. In that model, LCAT4 is transported and trapped inside autophagic bodies and requires LCAT3 to perform the initial hydrolyzing step, destabilizing these compartments and thus enabling LCAT4 to become active at acidic pH. A combination of both activities increases the disruption of autophagic bodies when LCAT3 and LCAT4 were expressed in yeast (Fig. 6), supporting the requirement and synergy of the proteins to efficiently process autophagic bodies in the vacuole. Finally, we note that plants lacking LCAT3 and LCAT4 are still able to perform autophagy degradation, albeit at a lower rate (Fig. 5). This indicates that additional phospholipases act redundantly (or compensate LCAT3/LCAT4) in this pathway.

With the exception of Atg15, a phospholipase B which controls this step of the pathway in yeast cells, little is known about the processing of autophagic bodies membranes across organisms. While yeast and plant cells show similar features in their lytic compartments, with large and unique vacuoles compared to the small and numerous lysosomes in mammals, LCAT3 and LCAT4 do not resemble Atg15, which has no sequence homologs in *Arabidopsis*. In contrast, they show sequence homologies with lysosomal phospholipases from human (PLA_2_G15; phospholipase A_2_ group XV) or from *C. Elegans* (LPLA-2) which have been involved in the degradation of intralumenal lysosomal material^32,33^. Nevertheless, the expression of LCAT3 in the vacuole of yeast cells can partially complement the absence of Atg15, suggesting that the two proteins may share some functional homologies. As mentioned above, recent work found that Atg15 has a phospholipase B activity, *i.e*., hydrolyze both acyl chains from phospholipids^9,10^. In contrast, LCAT3 and LCAT4 were found to have phospholipase A activities, hydrolyzing the acyl chain in *sn-1* and *sn-2* positions of phospholipids, respectively^19,27^. Whether and how the combined action of LCAT3 and LCAT4 could represent a functional analogy with the phospholipase B activity of Atg15 awaits future studies.

While the membrane of autophagic bodies needs to be broken off to ensure cargo delivery and degradation, this step must be tightly regulated in a spatiotemporal manner. In time, to efficiently process the large influx of autophagic bodies upon autophagy induction, and in space, to hydrolyze the membrane of autophagic bodies without affecting the homeostasis of the tonoplast which defects can lead to cell death^13^. How this regulation is achieved remains an open and critical question that our work only starts to address. Previous in vitro characterization of the phospholipase activity of LCAT3 and LCAT4 showed that both are only active in acidic pH^19,27^. This supports that LCAT3 and LCAT4 are inactive in the pH neutral cytosol even though LCAT4 is already associated with autophagy compartments. Once in the acidic sap of the vacuolar lumen, both proteins become active thereby spatially compartmentalizing the degradation of autophagic bodies. Other organelles of the cells (TGN, MVB) show an acidic pH, albeit more alkaline than that of the vacuole^12^. Depending on the trafficking route of LCAT3, we cannot exclude that LCAT3 may already become active prior to reaching the vacuolar lumen, and may therefore initiate membrane hydrolysis during autophagosome maturation as amphisomes (upon autophagosome/MVB fusion^6^) or in the recently characterized VAPV (VPS41-Associated Phagic Vacuoles) in which partial degradation was suggested^7^. Analyses of the amino-acid sequence of LCAT3 did not predict a specific signal peptide or a conserved vacuole sorting determinant. It is therefore difficult to speculate on the pathway used by LCAT3 to reach the vacuole. The canonical route for vacuolar resident proteins starts in the ER, passing first through the Golgi, then the Trans-Golgi-Network (TGN) and finally the MVB prior to reaching the vacuole lumen^34^. The accumulation of LCAT3 cytosolic puncta upon treatment with concanamycin A (Fig. 4b, d) could reflect its transient passing through this pathway as TGN maturation and MVB trafficking is partially blocked in these conditions^35^. In addition, our initial analyses to track LCAT3 trafficking indicate a partial co-localization of LCAT3-GFP with markers of the cis-Golgi (MEMB12^36^, 25%) or post-Golgi vesicles (VSR1^37^, 15%; Supplementary Fig. 7e–h). Further, a proportion of autophagosomes fuses with MVBs prior to their delivery to the vacuole^6^ which could be the point of contact between LCAT3 and the autophagy compartments. Supporting this idea, our analyses of the subcellular localization of LCAT3 found transient proximity between the cytosolic pool of LCAT3-marked vesicles and autophagosomes close to the tonoplast (Supplementary Movie 1). Future research should thus aim at elucidating the transport pathway of this protein to the vacuole and its relevance for autophagy degradation. In addition, within the vacuolar lumen, whether and how a specific localization of LCAT3 and/or LCAT4 at the membrane of autophagic bodies could spatially determine the site of their activity and thereby prevent tonoplast hydrolysis remain open mechanistic questions to be investigated. Recent work demonstrated that Atg15 can bind autophagic bodies membrane but not the tonoplast, explaining why this phospholipase is prevented to digest the vacuolar membrane in yeast event though the molecular mechanisms for such membrane specificity remain unknown^9^.

In addition to identifying the actors of autophagic bodies hydrolysis, our work here also finds that LCAT4 uses autophagy to traffic from the cytosol to the vacuole upon nutrient depletion (Fig. 2, Fig. 5). Based on these data, we propose an unsuspected conceptual leap in the field, showing that membrane hydrolysis is decided as early as its formation in the cytoplasm, during autophagosome biogenesis. This trafficking activity of autophagy puts the enzyme at the right place, *i.e*., at the membrane of autophagic bodies; at the right time, being transported inside the very vesicle that needs to be hydrolyzed; and at the same rate as their delivery inside the vacuole, to promote the efficient degradation of autophagic bodies. This data thus points to the somewhat unsuspected role of autophagy as a transport route in plants, prepping the vacuole to deal with the massive influx of autophagic bodies by packing up the hydrolytic enzyme(s) that will later be activated inside the vacuole and needed for its degradation and potentially that of its cargo. In that respect, the pathway that we identified here resembles that of the cytoplasm-to-vacuole targeting (Cvt) pathway, a selective type autophagy in *Saccharomyces cerevisiae*, which transports hydrolases to the vacuole and notably the aminopeptidase Ape1^38^. A similar transport route has never been identified in plants, where autophagy has solely been described as a degradation pathway^2^. Yet, a recent study described that, when overexpressed in *N. benthamiana*, the cysteine proteases VPEs from potato traffic to the vacuole using autophagy under carbon starvation^39^. This example diverges from the Cvt pathway, because even though autophagy is used as a mean of transport, VPEs do not promote cell survival but are instead involved in cell death through the destruction of the vacuolar membrane and the release of hydrolytic enzymes in the cytoplasm. Further, whether VPEs are specifically targeted and transported by autophagy or randomly sequestered inside autophagosomes during non-selective autophagy remains unknown. Further work is thus required to determine the prevalence of autophagy as a transport system for vacuole-bound hydrolases in plants.

In sum, our study unravels the trafficking function of autophagosomes in plants and characterizes a multi-component pathway required for the efficient and specific disruption of membranes of the autophagic bodies, thus highlighting this under-investigated step as a pivot for the completion of autophagy degradation.

## Methods

### Arabidopsis lines

All experiments were performed in *Arabidopsis thaliana* of the ecotype Col-0 as wild-type. The following transgenic lines were used as previously described: 35S::GFP-ATG8A^40^, pUBQ10::GFP-ATG8A^25^, pUBQ10::mCherry-ATG8F^41^, YFP-ATG18A^42^, *atg5-1*^43^, *atg7-2* transformed with pUBQ10::GFP-ATG8A^25^. TDNA insertion lines in *LCAT3 (lcat3;* SALK_035317) and *LCAT4 (lcat4*; SALK_147672.52.80) were obtained from the Nottingham *Arabidopsis* Stock Centre; homozygous lines were obtained by segregation and PCR genotyping using primers listed in Supplementary Data 1.

LCAT4-tagRFP, LCAT4-tagBFP and LCAT3-GFP plants were generated as following. The *LCAT4* or *LCAT3* coding sequence was PCR-amplified using primers listed in Supplementary Data 1 and cloned into the pDONR221 plasmid by Gateway BP reaction. The LCAT4^AIM^ sequence (with residues Y448, V449, I450, L451 mutated to alanines) was amplified by overlapping PCR using primers listed in Supplementary Data 1, to introduce the corresponding mutations. The gene sequences were verified by sequencing. The resulting plasmids were used for multisite gateway LR cloning together with the promoter UBQ10 cloned in the pDONRP4-P1R and tagRFP, tagBFP or GFP cloned in the pDONR2RP3 resulting in the construct pUBQ10::LCAT4-RFP, pUBQ10::LCAT4-BFP, pUBQ10-LCAT4^AIM^-RFP and pUBQ10::LCAT3-GFP in the expression plasmid PH7m34GW (LCAT4) or pLOCK180-pFR7m34G (LCAT3). The constructs were verified by sequencing and then transformed into GFP-ATG8A expressing plants for LCAT4-RFP, into pUBQ10::GFP-ATG8A expressing *lcat3,4* for LCAT4-RFP and LCAT4^AIM^-RFP, into YFP-ATG18A expressing plants for LCAT4-BFP and in mCherry-ATG8F for LCAT3-GFP by floral dip. Homozygous lines containing single construct insertions lines were selected based on Hygromycin B resistance (PH7m34GW-LCAT4) or based on red seed coat under epifluorescent microscopy with a set of filters: excitation BP 540–580 nm, emission LP 593 nm (pLOCK180-pFR7m34G-LCAT3).

To generate *Arabidopsis* lines double knock-out for *LCAT4* and *LCAT3*, *lcat3* and *lcat4* were crossed and the double homozygous line was selected by PCR using primers in Supplementary Data 1. To generate *Arabidopsis* lines expressing pUBQ10::GFP-ATG8A in the different genetic backgrounds, *lcat3*, *lcat4* or *lcat3 lcat4* were crossed with pUBQ10::GFP-ATG8A. The double and triple homozygous lines were selected by Hygromycin B resistance and PCR genotyping using primers listed in Supplementary Data 1.

For the generation of *amiRNA:lcat4* and *amiRNA:lcat3* lines, artificial microRNA sequences specific to the *LCAT4* gene (AT4G19860) or *LCAT3* gene (AT3G03310) were designed using the software WMD3 Web MicroRNA Designer. *amiRNA:lcat4* or *amiRNA:lcat3* precursor fragments were PCR amplified from the pRS300 plasmid as previously described^44^ using specific primers listed in Supplementary Data 1. PCR products were cloned into the pENTR/D-TOPO vector (pENTR Directional TOPO Cloning kit, Invitrogen). Clones were verified by sequencing and then transferred by gateway LR cloning into the destination vector PMDC7 which allows expression of the transgene under the control of β-estradiol. The constructs were transformed into *Arabidospis* 35S::GFP-ATG8A by floral dip; homozygous lines containing single construct insertions lines were selected based on Hygromycin B and Basta resistance.

### Growth conditions, autophagy induction and chemical treatments

Seeds were vernalized in water and darkness, at 4 °C, for 24 h to 48 h. The seeds were surface sterilized in 10% bleach for 30 min, and sown on Murashige and Skoog (MS) agar medium plates (4.4 g.L^−1^ MS powder including vitamins (Duchefa Biochemie M0222), 0.8% plant agar (Duchefa Biochemie, P1001), 1% sucrose (Merck Millipore, 84100) and 2.5 mM 2-(N-morpholino)-ethanesulphonic acid (MES, Euromedex EU0033), pH 5.7). Seedlings were grown vertically for 7 days, at 21 °C, under long-day conditions (16 h-light/8 h-dark photoperiod, 300 μE m^-^²s^−1^).

To induce autophagy, seedlings were transferred from MS plates to liquid media lacking nutrients as described hereafter for different times indicated in the figures. **(-N):** seedlings were transferred from MS plates into liquid MS-N medium (Murashige and Skoog liquid medium depleted for nitrogen: Murashige and Skoog micronutrient salts (Sigma, M0529), 3 mM CaCl_2_ (Sigma, C5670), 1.5 mM MgSO_4_ (Euromedex, P027), 5 mM KCl (Sigma, P9333), 1.25 mM KH_2_PO_4_ (Sigma P5655), 0.5 % (w/ v) D-mannitol (Sigma M9647), 3 mM MES, pH 5.7). **(-NC):** seedlings were transferred into liquid MS-N medium and incubated in darkness (wrapped in aluminum foil) for different times, as indicated in the figures. As control, for rich conditions (+ **N**), seedlings were systematically transferred from MS plates to full liquid MS medium (4.4 g.L^−1^ MS powder including vitamins, 1 % sucrose, and 2.5 mM MES, pH 5.7) under normal light conditions. Alternatively, autophagy was induced using the TOR inhibitor AZD-8055 (AZD, MedChemExpress, HY-10422) at concentration ranging from 1 μM (in Supplementary Fig. 3c) to 5 μM (in Supplementary Fig. 8) in liquid +N medium for the indicated times. BCECF (B1170, Thermofisher) was used to stain the vacuolar lumen^45^. Seedlings were transferred in +N, -NC or +AZD liquid medium for 2 hours prior to the addition of BCECF at 10 µM in the same medium in the dark for 1 h. To block autophagosome formation, seedlings were treated with Wortmannin (Sigma-Aldrich, 681675) for 3 h by transferring plants in liquid -NC medium + 1 μM Wortmannin. Concanamycin A (1 μM; Sigma-Aldrich, C9705) was used as previously described as an inhibitor of vacuole acidification to accumulate autophagic bodies in the vacuole^20^ by transferring plants in liquid -NC medium + CA for the indicated times. For all chemical treatments, DMSO (dimethyl sulfoxide, Sigma D8418) was used as vector control (untreated plants) in the same conditions (volume, time, type of medium) than the treated plants. To induce the expression of the artificial micro-RNA against *LCAT4* or *LCAT3*, seedlings were transferred in liquid medium containing 10 μM of β-estradiol (Sigma Aldrich, E2758) prior to autophagy induction. The incubation time varied from 8 to 16 h according to the experiments and are indicated in the respective figures.

### Structural analysis using AF3

AlphaFold 3 (https://www.alphafoldserver.com), developed by DeepMind, is an advanced AI platform for predicting protein structures. Protein sequences analyzed in this study were obtained from UniProt. The selected sequences were then submitted to the AlphaFold prediction module for detailed structural evaluation. The align command in UCSFChimera (version 1.17) was employed to superimpose the AF3 predictions onto known structures and to show the confidence score of the AF3 predictions using the local distance difference test (pLDDT) scores on the lDDT-Ca metric. pLDDT corresponds to the model’s predicted score on the lDDT-Cα metric^21^ and PAE plots provide valuable information about the reliability of relative position and orientations of different domains. PAE heatmap were designed using https://subtiwiki.uni-goettingen.de/v4/paeViewerDemo^46^.

### In vitro immunoprecipitation

All recombinant proteins were produced using *E. coli* strain Rosetta2 (DE3) pLysS. AIM wt or AIM mut were used as previously described^47^. ATG8A^ADS^ = ATG8A(Y50A,L51A). Transformed cells were grown in 2xTY media supplemented with 100 μg/mL Spectinomycin at 37 °C to log phase (OD_600_ 0.6–0.8), followed by induction with 300 μM isopropyl β-D-1-thiogalactopyranoside (IPTG) and incubation at 18 °C overnight. Cells were harvested by centrifugation for 10 min, 1500 x *g* at 4 °C, supernatant was discarded, and pellets were stored at –20 °C until further use. Bacterial pellets were resuspended in 5 mL Resuspension Buffer (100 mM NaPi pH 7.2, 300 mM NaCl, 1 mM DTT) supplemented with 1x cOmplete Protease Inhibitor Cocktail tablet (Roche) and 1 μg/mL Benzonase. Cells were lysed twice by 1–3 s on-off sonication cycles at 30% amplitude (Vibra Cell VCX750, Sonics & Materials) and cleared by centrifugation for 30 min, 21,130 x *g* at 4 °C in a top-table centrifuge. 5 µL of glutathione magnetic agarose beads (Pierce Glutathione Magnetic Agarose Beads, ThermoFisher Scientific) were equilibrated by washing them with IP buffer (100 mM NaPi pH 7.2, 300 mM NaCl, 1 mM DTT, 0.01% (v/v) IGEPAL® CA-630). Normalized *E. coli* clarified lysates were mixed and the final reaction volume was brought to 1 mL with IP Buffer. Reactions were added to the washed beads and incubated on an end-over-end rotator for 1 hour at 4 °C. After incubation, beads were washed 5 times in 1 mL wash buffer. Bound proteins were eluted and denatured in 50 µL 2X SDS Laemmli buffer (2% SDS, 10% glycerol, 5% 2-β-mercaptoethanol, 0.002% Bromophenol blue and 0.0675 M Tris HCl, pH 6.8) for 5 min at 95 °C prior to Western blotting. For Western blotting, the indicated total protein amount was loaded on 4–20% Mini-PROTEAN TGX precast SDS-PAGE gels (Bio-Rad) and blotted on nitrocellulose membranes (Bio-Rad) using the semi-dry Trans-Blot Turbo Transfer System (Bio-Rad). Membranes were blocked in TBS-T (10 mM Tris-HCl pH 7.5, 150 mM NaCl, 0.1% Tween 20) + 5% skimmed milk at room temperature for 1 h. After blocking, membranes were incubated with the respective primary antibody diluted in blocking buffer either for 1 h or overnight at 4 °C. The primary antibody, an anti-MBP (Sigma Aldrich, # M1321) at a dilution of 1/5000, was recovered, and membranes were washed 5 times for 5 min with TBS-T before incubation with the respective secondary antibody, an Anti-Mouse IgG-HRP Conjugate (Bio-Rad, #1706516) at 1/5000 or Anti-GST HRP-Conjugate (GE Healthcare, # RPN1236) at 1/5000, for 45 min at room temperature. The immune reaction was developed using Pierce™ ECL Western Blotting Substrate (ThermoFisher) and detected with iBright Imaging System (Invitrogen).

### In vivo immuno-isolation and co-immunoprecipitation

Data from Fig. 1a, were obtained from immuno-isolations of ATG8-labeled compartments performed in ref.^16^. In Fig. 5, in vivo co-immunoprecipitations were performed using about 100 seedlings of each genotype (GFP-ATG8A expressing *lcat3,4* mutant non complemented, complemented with LCAT4-RFP or complemented with LCAT4^AIM^-RFP). Seedlings were grown in MS media for 7 days prior to autophagy induction in MS-NC liquid medium for 1 h. Seedlings were grinded with 700 μL of EDTA-free buffer (10% glycerol, 25 mM Tris-HCl, 150 mM NaCl, 0.1 mM TCEP, 0.1% IGEPAL, 10 μM ZnCl_2_ and EDTA-free Protease Inhibitor Cocktail mini complete (Sigma Aldrich)) with 2% PVPP. Lysates were centrifuged twice at 17,000 x *g* at 4 °C for 10 min to eliminate debris. 500 μL of the resulting cleared lysates were incubated for 1 h at 4 °C with gentle rotation with 20 μL GFP-Trap Magnetic Agarose beads (ChromoTek, previsouly washed three times with EDTA-free buffer without TCEP). Beads were washed five times after the incubation with lysates prior to elution with 60 μL 2x Laemmli buffer, denaturation at 55 °C for 20 min and Western blot analyses with the antibodies indicated in Fig. 5i.

### Phenotypic assays

For germination assays, seeds were vernalized for two days in water prior to being sown on MS ½ + 1% sucrose. 20–30 seeds of each genotype were analyzed per plate for a total of 60 seeds per genotype. Plates were placed horizontally at 21 °C, under long-day conditions (16 h-light/8 h-dark photoperiod, 300 μE m^-^²s^−1^). At the indicated times, seed germination was assessed qualitatively by examining the tegument of the seed and the emergence of the radicle. Results are expressed as the percentage of germinated seeds over total seeds per independent plate. The experiment was performed in two biological replicates with lot#1 (each genotype in the Col-0 background) and lot#2 (each genotype expressing pUBQ10:GFP-ATG8A). For root length measurement, similar settings and conditions were used for plant growth in vitro. Each plate was imaged at 54 h, 72 h and 96 h after sowing and root length was marked. Root length was measured using the Fiji software (National Institutes of Health, USA, http://imagej.nih.gov/ij) using the aforementioned images. For comparison of vegetative growth and onset of senescence, seedlings of each indicated genotype were sown on 0.8 % plant agar plates containing full-MS powder including vitamins, 1% sucrose and 2.5 mM MES and then grown at 21 °C under an LD (16 h-light/8-h-dark) photoperiod. After 1 week, seedlings were transplanted to soil and grown at 21 °C under SD (8- h-light/16 h- dark photoperiod) conditions for either 6 or 12 weeks and imaged. To assess the effects of nutrient limitation on plant physiology, seedlings of each indicated genotype were grown for 1 week on MS plates in rich conditions as described above. Seedling were then transferred for 10 days to a solid minimal medium lacking sucrose and nitrogen (medium was prepared as in ref. ^48^, without sucrose) and wrapped with aluminum foil. After 10 days, plates were imaged and the color of the seedlings was assessed qualitatively. Nine independent plates containing about 20 seedlings of each genotype, grown and treated side by side, were analyzed. For each plate, the percentage of green seedlings was calculated as the number of mostly green seedlings over total seedlings.

### Microscopy analyses of live cells

All live confocal observations were performed on root epidermal cells of the transition zone and elongation to early differentiation zone, employing 7-day-old seedlings mounted in culture medium as indicated in the figures. Roots were kept under the microscope for no longer than 10 min in order to avoid secondary effects due to prolonged treatment times. Confocal images were acquired with a ZEISS LSM880 confocal system. Laser excitation lines for the different fluorophores were 405 nm for BFP, 488 nm for GFP, 514 nm for YFP and 561 nm for RFP or mCherry. Fluorescence emissions were detected at 420-470 nm for BFP, 490–597 nm for GFP, 520-555 nm for YFP, 580–650 nm for RFP and 561-633 nm for mCherry. In multi-labeling acquisitions, detection was in sequential line-scanning mode. Confocal microscope images were processed using the Zen Black Software (Zeiss) for intensity optimization. For puncta quantification and co-localization analyses in the cytosol, n number of images as indicated in the figure legends were taken in a minimum of 5 independent roots in B number of biological experiments as indicated in the figure legends. Puncta were counted manually in each image and each channel and root surface areas were quantified using Fiji to obtain the puncta/surface values. When multiple z-planes were imaged, the number of puncta and the surface of the area was quantified in each plane and averaged. For puncta quantification and co-localization analyses in the vacuole, a similar image acquisition and analytical pipeline protocol was followed with the exception that puncta were counted manually in selected cells where the vacuole was clearly discernable from cortical cytosol (using the GFP-ATG8A signal as a marker of the cytosol). The surface of each analyzed cell was quantified to obtain the puncta/surface values. Co-localization between ATG8 and LCAT3 or LCAT4 was assessed manually comparing the position of puncta in each channel, overlapping signals indicated colocalization. Co-localization was confirmed on selected puncta, by comparing the maximum intensity of signal in each channel plotted along a line using the plot profile tool of Fiji. To measure the vacuole/cytosol signal intensity ratio in Fig. 2c, cells (number indicated in the figure legend) where the vacuole was clearly discernable were selected in 5-11 independent roots. Signal intensity was quantified using Fiji, either at the cell periphery or inside the vacuolar lumen, in a delimitated region of interest of the same surface for each location. In Fig. 4c, high-zoom time series images of vacuoles were analyzed using the TrackMate plugin^49^. Tracking was done on the mCherry-ATG8 channel, while LCAT3 co-localization events were manually identified. Images of localization of CPY-LCAT3-GFP and CPY-LCAT4-GFP in yeast were acquired using a CCD CoolSnap HQ2 camera mounted on a ZEISS AxioImager epifluorescence microscope. GFP was observed using a dedicated filter setup (Excitation 472/30, Emission 520/55).

### Immunocytochemistry and confocal laser scanning microscopy

For immunocytochemistry in Supplementary Fig. 7e, g, 7-day-old seedlings were placed in the dark, in liquid -NC with 1 µM concanamycin A for 3 h. Seedlings were rinsed first in water, then in MTSB [50 mM PIPES (Merck, P1851), 5 mM EGTA, 5 mM MgSO_4_ pH 7 with KOH]. Whole-mount immunolabelling of roots was performed immediately after as described in ref.^50^. In brief, seedlings were fixed for 1 h at room temperature in 4% paraformaldehyde (Agar Scientific, AGR1026) dissolved in MTSB and washed three times with MTSB. Roots were cut on superfrost slides (Menzel Gläser, Germany) and dried at room temperature. Roots were then permeabilized with 2% Driselase (Merck, D9515), dissolved in MTSB for 30 min at room temperature, rinsed four times with MTSB, and treated for 1 h at room temperature with 10% DMSO (Merck, D8418) + 3% Igepal CA-630 (Merck, I3021) dissolved in MTSB. Unspecific sites were blocked with 5% normal donkey serum (NDS; Merck, D9663) in MTSB for 1 h at room temperature. Primary antibodies, in 5% NDS/ MTSB, were incubated overnight at 4 °C and then washed four times with MTSB. Secondary antibodies, in 5% NDS/MTSB, were incubated for 1 h at room temperature and then washed four times with MTSB. Primary antibodies were diluted as follows: rabbit anti-MEMBRIN12 1:300 (MEMB12, kind gift of Patrick Moreau and Yohann Boutté, University of Bordeaux, France), rabbit anti-VSR1 1:300 (AGRISERA, AS20 4407). Dilution of secondary antibody Atto 647N-coupled goat anti-rabbit IgG (Merck, 40839) was 1:300. The Intavis InsituPro VSi automated system was used to perform automated immuno labelling. High resolution Airyscan imaging of GFP, mCherry and Atto 647 N were performed using the ZEISS LSM880 confocal microscope (Carl Zeiss AG, Oberkochen, Germany) equipped with a Plan-Apochromat 63x/1.4 Oil DIC UV-VIS-IR M27 objective and a super-resolution AiryScan module with a band-pass filter 465–505 nm and a long pass filter 525 nm. Laser excitation lines for the different fluorophores were 488 nm for GFP, 561 nm for mCherry and 633 nm for Atto 647 N. Images were acquired with the Airyscan detector. Scanning was performed with a pixel dwell of 2.65 μs and no averaging. In multilabelling acquisitions, detection was in sequential frame-scanning mode. Image analysis was performed using ZEN lite 2.6 2018 (Zeiss) and ImageJ software. Image and co-localization analysis were performed using ZEN lite 2.6 2018 (Zeiss) and Fiji software as described for live imaging. In total, 592 and 622 LCAT3 puncta, originating from five distinct roots, were quantified to assess their respective co-localization rates with MEMB12 and VSR1.

### High pressure freezing and freeze substitution

5-days old *Arabidopsis* seedlings expressing LCAT3-GFP were placed in liquid full MS (+ N) with 1 µM AZD-8055 and 1 µM concanamycin A during 4 h. Root tips were cut and place in copper carriers filled with liquid MS + 20% bovine serum albumin (w/v) as cryoprotectant. Samples were frozen using Leica EM-ICE high pressure freezer and transferred at −90 °C into the Leica AFS 2 freeze-substitution device and were incubated in a cryosubstitution mix containing 0.1% uranyl acetate (w/v) in pure acetone for 30 h. The temperature was then raised progressively to −50 °C, at 3 °C/h. Cryosubstitution mix was removed and washed three times with pure acetone and three times in pure ethanol. Samples were embedded in HM20 Lowicryl resin: HM20 25% v/v, 50% v/v (2 h each), 75% v/v (overnight), 100% (twice for 2 h), and a last 100% for 8 h. The polymerization was done under UV light for 24 h at −50 °C followed by 12 h at +20 °C.

### EM immunolabeling and EM imaging

Ultrathin sections of 80 nm were made using Leica EM-UC7 ultramicrotome and harvested on parlodion coated grids. Immunogold labelling against GFP was performed using polyclonal anti-GFP primary antibody (Torrey pines TP-401) diluted at 1:100. Antibody binding was detected with 10 nm gold-conjugated goat anti-rabbit antibodies diluted at 1:30 (Aurion GAR EM10). Transmission electron microscopy observations were carried out on a FEI TECNAI Spirit 120 kV electron microscope equipped with Gatan Rio 16 camera and serialEM acquisition software^51^.

### Transient transformation of Arabidopsis cotyledons

For transient expression in cotyledons, seeds of WT, *atg5-1* or pUBQ10::mCherry-ATG8F were grown on MS plates in conditions described above, for 4 days. Transformation was performed using *Agrobacterium tumefaciens* C58C1Rif^R^ strain harbouring both the pMP90 plasmid and the pUBQ10::LCAT3-GFP plasmid. Four days after germination, *A. tumefaciens* suspension in MS-Glucose liquid medium [0.21% MS (Duchefa Biochemie, M0222), 2% glucose (Euromedex, UG3050), 0.39% MES (Euromedex EU0033), 0.05% Tween20 (Merck P1379), 200 mM acetosyringone (3’,5’-dimethoxy-4’hydroxyaceto-phenone; Merck D134406), pH 5.7] was used to transiently transform the cotyledons. For that, seedlings were incubated for 40 min at room temperature with a suspension of *A. tumefaciens* expressing LCAT3-GFP at 1 OD_600 nm_. The suspension was then removed and the 6-well culture plate were left in 16 h light/8 h darkness until the seventh day after germination. Autophagy was then induced by transferring seedlings from MS 6-well culture plates into liquid MS medium containing 5 µM AZD-8055 (MedChemExpress, HY-10422) for TOR inhibition. Concomitantly with autophagy induction, chemical treatment was applied with 1 µM Concanamycin A (Santa Cruz Biotechnology INC, sc-202111A) for V-ATPase activity inhibition. Seedlings were then grown in these conditions in 16 h light/8 h darkness for another 24 h. Confocal laser scanning microscopy was performed using a Zeiss LSM880 microscope (Carl Zeiss AG, Oberkochen, Germany) equipped with a Plan Apochromat ×40, 1.3 Oil DIC UV-VIS-IR M27. Cotyledons were cut and mounted with MS liquid medium containing 5 µM AZD-8055 (MedChemExpress, HY-10422) and 1 µM Concanamycin A (Santa Cruz Biotechnology INC, sc-202111A). Laser excitation lines for the different fluorophores were 488 nm for GFP and 561 nm for mCherry. Fluorescence emissions were detected at 490–553 nm for GFP and 563–651 nm for mCherry. Z-stack acquisitions at 1 µm intervals were performed. Detection was in sequential line-scanning mode with no averaging and a scanning pixel dwell of 1.81 µs. Image analysis was performed using ZEN lite 2.6 2018 (Zeiss) and ImageJ software.

### Protoplasts isolation and transformation

Mesophyll protoplasts were isolated as described in ref. ^52^ from true leaves of 7-week-old plants of Col-0 wild-type, *atg5-1* and spL-RFP^53^ backgrounds. Protoplasts were transformed following the protocol described in ref. ^52^, placed in a 24-well plate with microscopy-grade coverslip bottom (Cellvis) and incubated for 17 h under long day conditions, after which the 24-well plate was mounted on an inverted Zeiss CLSM800 and imaged to confirm transgene expression. AZD8055 and Concanamycin A were added to protoplast medium to the final concentrations of 5 µM and 0.5 µM respectively, and the protoplasts were incubated under long day conditions for another 24 h prior to imaging.

### Western-blot analyses

To assess autophagy flux, we performed the GFP-ATG8 processing assay: 7-day-old seedlings were transferred from full MS plates into rich MS liquid medium (+ NC), -N liquid medium (-N) or -NC liquid medium (-NC) and total proteins were extracted after treatments. Whole seedlings were frozen in liquid nitrogen, disrupted using a TissueLyser (Qiagen), and homogenized in the following buffer: 100 mM Tris pH 7.5, 200 mM NaCl, 1 mM EDTA, 2% β-mercaptoethanol, 0.2% Triton 100X, 1 mM PMSF and antiprotease mix (P9599 Sigma). Homogenates were centrifuged at 1600 × g for 20 min at 4 °C and twice at 1600 × *g* for 10 min at 4 °C, transferring only the supernatant at each stage. The protein concentration of each sample was determined using Bio-Rad Protein Assay Dye Reagent Concentrate, (BioRad, #5000006) and measuring sample absorption at 595 nm. Equal amounts of proteins were denatured using Laemmli buffer at 55 °C for 15 min and loaded on acrylamide gels. After migration on 12% SDS-PAGE (TGX Stain-Free FastCast Acrylamide kit, BioRad), equal protein quantity per lane was verified by stain-free activation of the loaded gel. After transfer to nitrocellulose (BioRad, #1704270), membranes were incubated with an anti-GFP mouse antibody (Roche, #11814460001) at a dilution of 1/10,000 for 35S:GFP-ATG8A expressing plants (Supplementary Fig. 4f, 7). For pUBQ10:GFP-ATG8A plants, blots were cut to separate the GFP-ATG8 bands (dilution at 1/5000) from the GFP bands (dilution at 1/10,000) to allow quantitative analyses of each band without saturation of the free GFP signal (Figs. 3–5). Peroxidase activity coupled to the Goat anti-Mouse antibody at a dilution of 1/10,000 (Bio-Rad, #1706516) was revealed using Clarity Max Wester ECL (Biorad #170562) and detected with the Chemidoc MP Imaging system (Bio-Rad). Blots were imaged at different exposition times, long enough to observe clear distinct bands but prior to pixel saturation. Blot images were analyzed using the Fiji software; band intensities were calculated using the plot surface areas functionality of the software. The intensities of the free-GFP band and the GFP-ATG8 band were then used to calculate the relative GFP/GFP-ATG8 ratios and compare it to that of WT which was set to 1 in each independent experiment.

To assess the level of LCAT4 upon nutrient starvation, 7-day-old plants expressing pUBQ10::LCAT4-RFP were transferred in (+ N) or (-NC) liquid medium for 3 h. Total proteins were extracted, quantified and denaturated as describe above. Equal amounts of total proteins were loaded on 12% SDS-PAGE gels and the level of LCAT4-RFP was analyzed by immunoblotting using anti-RFP antibody (Agrisera, AS153028; 1/1000). Peroxidase activity coupled to the Goat anti-Rabbit secondary antibody (1/10,000; Biorad, #1706515) was used for revelation. Equal protein quantity per lane was verified by stain-free activation of the loaded gel. Blot images were analyzed using the Fiji software as described above and the amount of LCAT4 was normalized to that of total protein measured in the stain free. To assess the level of LCAT4 after prolonged starvation, the same experiments were performed by transferring plants in (+ N), (-NC) and (-NC + CA) liquid medium for 8 h.

### RT-QPCR

Total RNA was extracted using the RNeasy mini kit (Qiagen). To eliminate genomic DNA contamination, an additional DNase treatment was performed according to the RNeasy kit instruction with the DNA removal kit (Invitrogen). One microgram of total RNA was reverse transcribed into cDNA in a 20 μL reaction mixture using the Superscript IV reverse transcriptase enzyme (Invitrogen). cDNA levels were then analyzed using the iQ™ Sybr Green supermix (BioRad) on the iQ iCycler thermocycler (BioRad) with the gene-specific primers listed in Supplementary Data 1. The thermocycling program consisted of one hold at 95 °C for 3 min, followed by 40 cycles of 15 s at 95 °C and 30 s at 58 °C, and a melt curve from 65 °C to 90 °C with an increment of 0.5 °C all 5 s. After completion of these cycles, melting-curve data were then collected to verify PCR specificity, contamination, and the absence of primer dimers. The transcript abundance in samples was determined using a comparative threshold cycle method. The relative abundance of the reference mRNAs of *ACTIN 2/8*, *AT4G33380* or *SAND* was determined in each sample and used for normalization, according to the experiment.

### Yeast strains, expression, media and culture

Yeast strains used in this study are listed in Supplementary Data 2. To generate the PYES3-LCAT3, PYES3-LCAT4 and pYES2-LCAT4 plasmids, the coding sequences of the corresponding genes were cloned from the pDONR221 (see *Arabidopsis* lines) into the PYES3 or pYES2 plasmid (both harboring the GAL1 promoter and either TRP or URA auxotrophic genes, respectively) using LR gateway cloning. To generate pYES3-CPY-LCAT3, pYES3-CPY-LCAT4 or pYES2-CPY-LCAT4, a DNA fragment encoding the first 50 amino acids of the CPY protein (CPY^1–50^; from the *PRC1* gene) was PCR amplified from *S. cerevisiae* total genomic DNA using the primers listed in Supplementary Data 1. The forward primer was flanked with the Attb1 sequence while the reverse primer was flanked with a DNA fragment consisting of the first 26 nucleotides of *LCAT4* or first 20 nucleotides of *LCAT3*. The coding sequence of *LCAT3* and *LCAT4* were PCR amplified using primers listed in Supplementary Data 1, with the forward primer flanked with the last 24 nucleotides of CPY^1–50^ and the reverse primers flanked with Attb2 sequence. Then, the CPY^1–50^ fragment and LCAT3 or LCAT4 coding sequence were used as template and fused using overlapping PCR to generate CPY^1–50^-LCAT3 or CPY^1–50^-LCAT4. The resulting DNA fragments were cloned into the PDONR221, sequence verified and then transferred into the PYES3 or pYES2 plasmid using gateway cloning. pYES3-CPY-LCAT3^S177A^ and pYES2-LCAT4^S182A^ was generated similarly after performing site directed mutagenesis to change serine 177 (in LCAT3) or serine 182 (in LCAT4) to an alanine by overlapping PCR using primers listed in Supplementary Data 1.

In Fig.6a-c, to express LCAT3, LCAT4, CPY-LCAT3, CPY-LCAT4 or CPY-LCAT3^S177A^, SSY12 yeast cells (see Supplementary Data 2) were transformed with the corresponding plasmids and transformants were selected on synthetic minimal medium lacking tryptophan (SMD; 0.67% yeast nitrogen base, 2% glucose, 0.2% drop out -Trp). As controls, SSY12 or SEY6210 yeast cells were transformed with the empty PYES3 plasmid. To induce the expression of the transgenes, yeast cells were grown overnight in SMGal lacking tryptophan (0.67% yeast nitrogen base, 2% galactose, 0.2% drop out). The following morning, cultures were diluted back to 0.2 OD and yeast cells were grown until mid-log phase (OD 0.6-1). 1 OD of each culture was collected and total protein were extracted as previously described^54^. Equal amounts of proteins were loaded on 12% SDS-PAGE gels and immunoblot analyses were performed using nitrocellulose membranes, the Ape1 primary antibody^55^ (1/10,000) and a peroxidase-coupled Goat anti-Rabbit secondary antibody (1/10,000; Biorad, #1706515).

In Fig. 6d-g, to construct GFP-AtATG8A expressing cells, the coding sequence of GFP and that of AtATG8A were PCR amplified and fused using overlapping PCR using primer listed in Supplementary Data 1. The resulting transgene was digested with PacI and AscI and ligated into the pFa6a-HIS plasmid^56^. Endogenous Atg8 was disrupted and replaced by GFP-AtATG8A-HIS at the Atg8 locus using a standard method for chromosomal tagging^56^ by amplifying GFP-AtATG8A-HIS from the previously constructed pFa6a:GFP-AtATG8A-HIS plasmid using primers listed in Supplementary Data 1and transforming either WT cells (SEY6210) or *atg15Δ* cells (TKYM108), see Supplementary Data 2. Transformants were selected on minimal medium lacking histidine (SMD; 0.67% yeast nitrogen base, 2% glucose, 0.2% drop out -His). Replacement of endogenous Atg8 by GFP-AtATG8A was verified by PCR using primers listed in Supplementary Data 1. The resulting WT cells (YAB610) or *atg15Δ* cells (YAB613) expressing GFP-AtATG8A were used for further yeast transformation to express either CPY-LCAT3 (cloned in pYES3), LCAT4 or CPY-LCAT4 (cloned in pYES2) and a combination of CPY-LCAT3 (cloned in pYES3), LCAT4 (cloned in pYES2), LCAT4^S182A^ or empty plasmids as listed in Supplementary Data 2. Yeast cells transformed with pYES3 plasmids were selected on minimal medium lacking tryptophan; cells transformed with pYES2 plasmids were selected on minimal medium lacking uracil and cells transformed with pYES2 and pYES3 plasmids were selected on minimal medium lacking both tryptophan and uracil (SMD; 0.67% yeast nitrogen base, 2% glucose, 0.2% appropriate drop out). To induce the expression of the transgenes, yeast cells were grown overnight in SMGal lacking either tryptophan, uracil or both (0.67% yeast nitrogen base, 2% galactose, 0.2% drop out). The following morning, cultures were diluted back and yeast cells were grown until mid-log phase (OD 0.6-1) prior to inducing autophagy by transferring cells into SGal-N medium (0.17% yeast nitrogen base without amino acids, containing 2% galactose). In Fig. 6d, f; cells were starved for 4 hours; in Fig. 6e, g, cells were starved for 10 hours. 1 OD of each culture in +N or -N condition was collected. Protein extraction, immunoblot and GFP-ATG8 processing were performed as previously described^54^. Equal amounts of proteins were loaded on 12% SDS-PAGE gels and immunoblot analyses were performed using nitrocellulose membranes, the GFP primary antibody (1/10,000; Roche, #11814460001) and a peroxidase-coupled Goat anti-Mouse secondary antibody (1/10,000; Bio-Rad #1706516).

FM 4-64 staining of yeast vacuoles were performed as previously described^57^ with minor modifications. TKYM108 cells transformed with CPY-LCAT3-GFP or CPY-LCAT4-GFP were grown overnight in SMGal lacking tryptophan, diluted back to 0.2 and grown in YPGal until exponential phase. A final concentration of 5 µM FM 4-64 (Invitrogen, #T13320) was used to label the cells in YPGal for 20 min at 30 °C followed by a chased for 2 h in YPGal and directly imaged or starved for 10 h in SGal-N and imaged using the ZEISS LSM880 confocal system as described above.

### Statistical analyses

Statistical analyses were performed using GraphPad Prism 11 (GraphPad Software, La Jolla, CA, USA) with tests indicated in the figure legends. *P*-values are as follows: *P*-value > 0.05 (non-significant, ns), *P < 0.05, **P < 0.01 and ***P < 0.001.

### Reporting summary

Further information on research design is available in the Nature Portfolio Reporting Summary linked to this article.

## Supplementary information

Supplementary Information

Descriptions of Additional Supplementary Files

Supplementary Data 1

Supplementary Data 2

Supplementary Movie 1

Reporting Summary

Transparent Peer Review file

## Source data

Source Data

## Data Availability

Source data are provided in the Source Data file, except raw images of confocal microscopy which will be provided by the corresponding author upon request.  Source data are provided with this paper.
