# Supplementary material for: A dual phospholipase system instructs membrane hydrolysis during the final stages of plant autophagy
Source: Nat Commun. 2026 May 14;17:6444. doi: 10.1038/s41467-026-73116-x (PMC13377104; doi:10.1038/s41467-026-73116-x)
Supplement: Supplementary file 1 — Supplementary Information [file 41467_2026_73116_MOESM1_ESM.pdf]

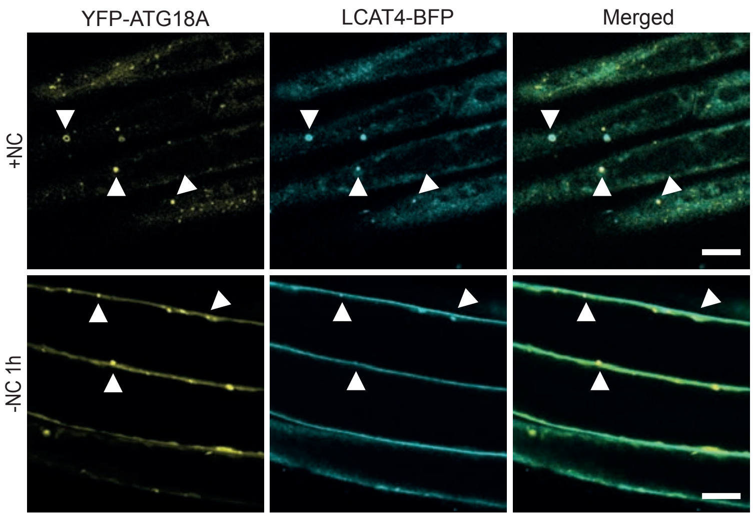

Supplementary Figure 1. LCAT4 co-localizes with YFP-ATG18A.
Representative confocal images of 7-day-old seedlings co-expressing YFP-ATG18A and LCAT4-BFP. Plants were placed in liquid MS medium in rich condition (+NC) or deprived of nutrients for 1h (−NC 1h). The experiment was repeated two times (+NC) or three times (-NC) with similar results. Arrowheads indicate colocalization. Scale bar, 10 µm.

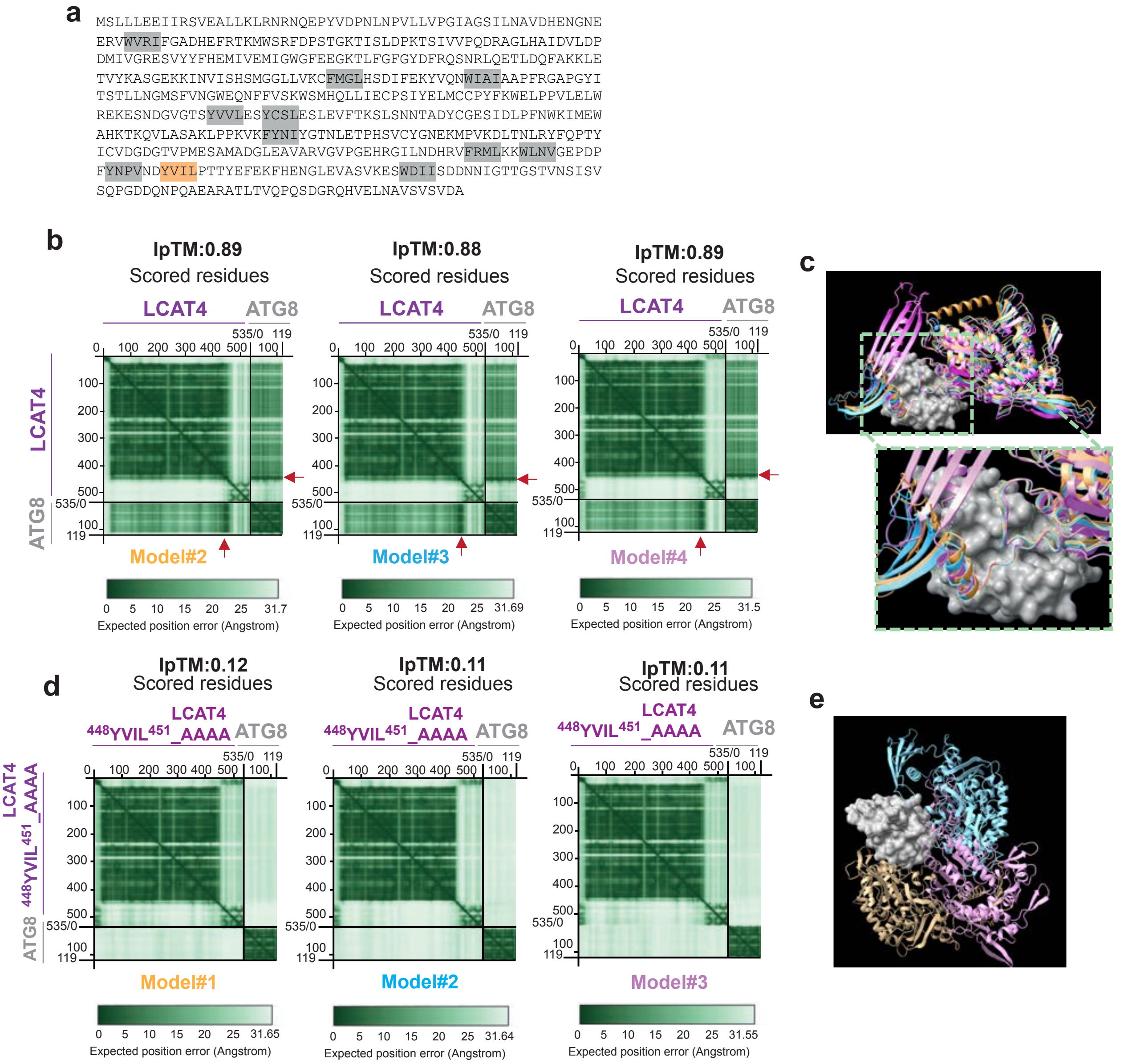

Supplementary Figure 2. Prediction of the LCAT4-ATG8 interaction.

a Analysis of the amino acid sequence of LCAT4 shows several [W,Y,F][X][X][L,I,V] motifs, highlighted in grey. The AlphaFold3 predicted AIM domain ^{448}YVIL^{451} is highlighted in orange. **b-e** Predicted aligned error (PAE) plots and graphic representation of three models based on AlphaFold3 multimer modeling of the LCAT4-ATG8 interaction using WT LCAT4 (**b,c**) or a mutated version of LCAT4 where the four residues of the predicted AIM domain where replaced by alanines (^{448}YVIL^{451}_AAAA, **d,e**). Units: amino acid residues; light green to dark green: expected position error in angstroms. IpTM are indicated at the top of each PAE plots. Three best-scoring models from AlphaFold3 show almost identical binding interfaces between WT LCAT4 and ATG8 (**b,c**). The LCAT4-ATG8 interaction interface is shown by the arrows in each protein (**b**). Neither of the three models from AlphaFold3 show an interface between LCAT4^{448}YVIL^{451}_AAAA and ATG8 (**d,e**).

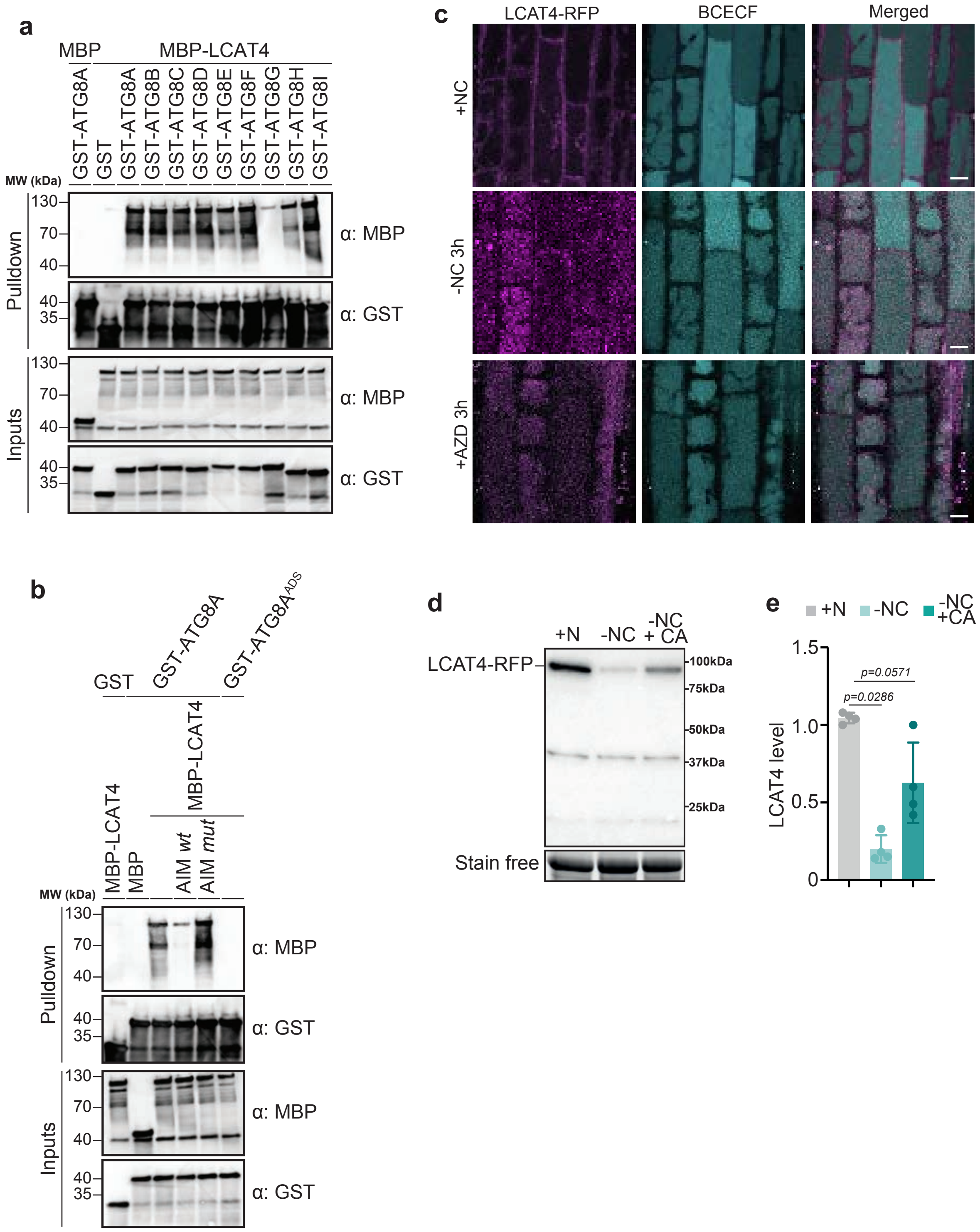

Supplementary Figure 3. LCAT4 binds all ATG8 isoforms *in vitro* and relocates to the vacuole upon autophagy inducing conditions.
a LCAT4 binds all ATG8 isoforms *in vitro*. Bacterial lysates containing recombinant protein were mixed and pulled down with glutathione magnetic agarose beads. Input and bound proteins were visualized by immunoblotting with anti-GST and anti-MBP antibodies. Representative images of n=2 independent experiments. **b** LCAT4 interacts with ATG8 in an AIM-dependent manner. ATG8A^{ADS}=ATG8A(Y50A,L51A). AIM *wt* and AIM *mut* peptides were added to a final concentration of 200 µM. Bacterial lysates containing recombinant protein were mixed and pulled down with glutathione magnetic agarose beads. Input and bound proteins were visualized by immunoblotting with anti-GST and anti-MBP antibodies. Representative images of n=2 independent experiments. **c** LCAT4 is found in the vacuole under autophagy induction conditions. Representative confocal images of Arabidopsis roots expressing LCAT4-RFP in the transition zone. Plants were imaged in nutrient rich condition (+NC), after 3 hours in nutrient starvation (-NC) or +NC with the addition of AZD8055 (AZD; 1 µM). Vacuoles were stained with BCECF/AM (BCECF, 10 µM). The experiment was repeated in 8 (+N), 15 (-NC) and 7 (+AZD) independent roots with similar results. Scale bar, 10 µm. **d-e** LCAT4 is partially degraded upon prolonged starvation. Detection of LCAT4-RFP by immunoblot from total proteins extracted from 7-day-old seedlings after 8 hours in liquid rich condition (+N) or nutrient starved liquid medium (-NC) +/- concanamycinA (-NC+CA). The experiment was repeated four times with similar results (**d**). The level of LCAT4 was detected, quantified and normalized by the loading control; all values are presented with average and SD, statistical differences were assessed using two-tailed Mann whitney tests (**e**).

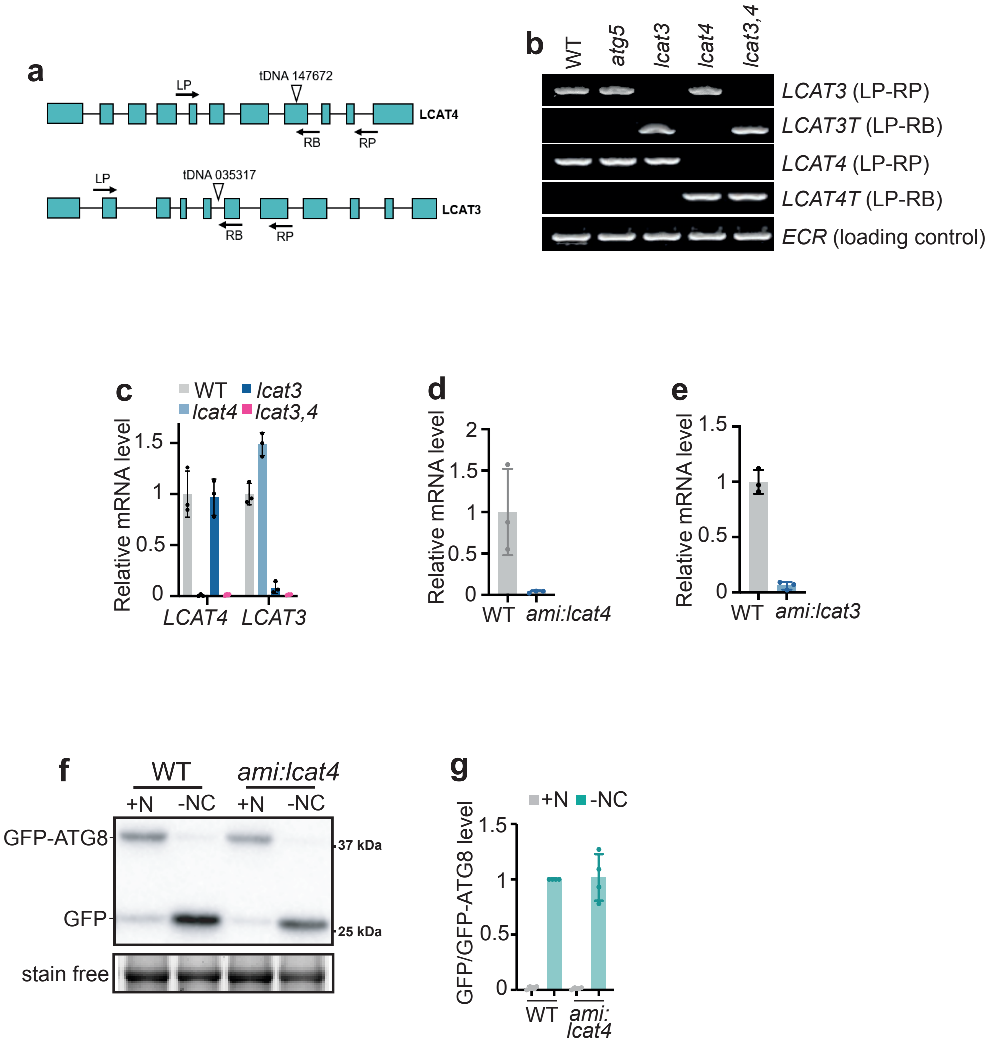

Supplementary Figure 4. Characterization of the *LCAT4* and *LCAT3* transgenic lines
a Representative schemes of T-DNA insertion in the *LCAT4* or *LCAT3* genes. Exons, thick squares; introns, thin lines. Positions of PCR primers for genotyping are indicated; gene-specific (LP, RP) and T-DNA specific (RB). **b** PCR genotyping of *lcat3*, *lcat4* and *lcat3,4* mutants compared to WT and *atg5* plants. *ECR* was amplified as control. The experiment was repeated three times with similar results. **c** *LCAT4* and *LCAT3* mRNA levels in *lcat3*, *lcat4* and *lcat3,4* mutants compared to WT (set to 1 in each experiment) in 7-day-old seedlings. Results represent the average ± SD of 3 replicates; normalized by the reference genes *SAND* and *ACTIN 2/8*. **d-e** Analyses of the transcripts in *amiRNA:LCAT4* (**d**) or *amiRNA:LCAT3* (**e**) lines showing a decrease in the expression of the target gene compared to WT plants. 7-day-old seedlings were treated with β-estradiol (10 μM) for 24 h in (**d**) or 8 h in (**e**). Results represent the average ± SD of 3 replicates; normalized by the reference genes *ACTIN 2/8* and *AT4G33380* in (**d**) or by *ACTIN 2/8* in (**e**). **f-g** Analysis of the autophagic flux in the *amiRNA:LCAT4* line. **f** Immunoblot analyses of GFP-ATG8A and its degradation product in WT or *amiRNA:LCAT4* plants expressing 35S:GFP-ATG8A. Seedling were transferred to either (+N) or (-NC) for 16 hours ± 10 μM of β-estradiol. Total proteins were extracted and analyzed with anti-GFP antibodies. Stain free images were used as loading control. The experiment was repeated four times with similar results. **g** Quantification of the ratio of GFP/GFP-ATG8A in (**a**) relative to that of WT plants in (-NC) condition which was set to 1 in each experiment. Results are presented as the average with values of distinct replicates; n=4 biological replicates.

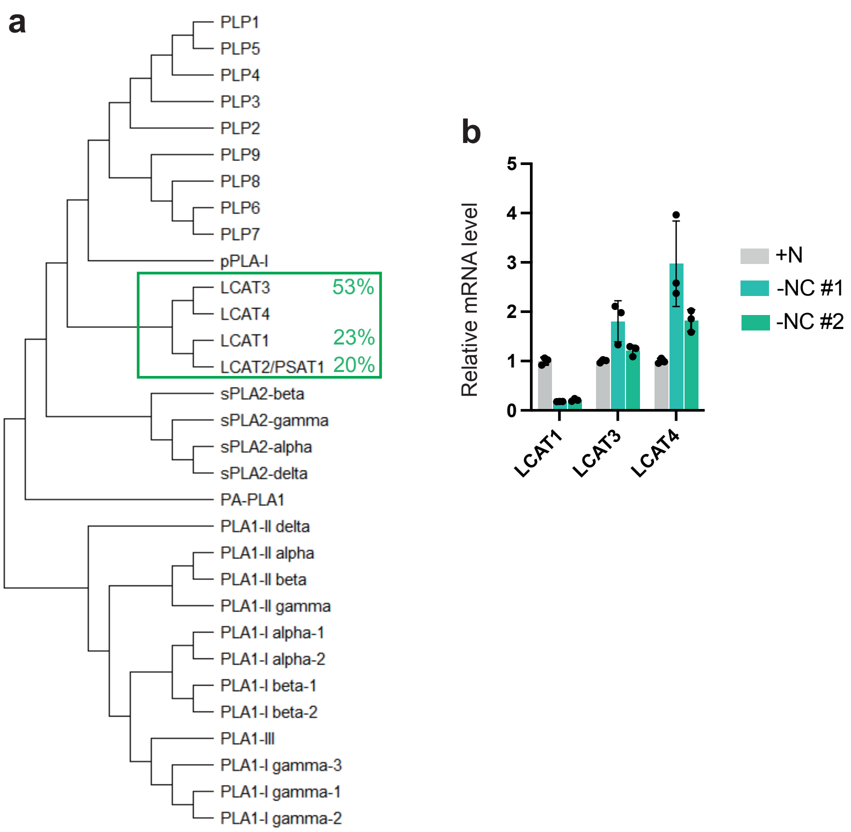

Supplementary Figure 5. LCAT1 and LCAT3 are homologs of LCAT4.

a Phylogenetic tree of Arabidopsis phospholipases. The amino acid sequences were aligned using the Clustal Omega algorithm; phylogenetic analyses were performed using MEGA11. Phylogenetic relationships between taxons were established using the maximum likelihood method and the robustness of the tree obtained was tested with a bootstrap of 1000 replicas. Percentage of identity between LCAT4 and the other LCAT members is indicated in green. **b** Relative abundance of the transcripts of *LCAT1*, *LCAT3* and *LCAT4* in 7-day-old seedlings placed in rich condition (+N, liquid medium) or deprived of nutrients (-NC, liquid medium) for 6h. Levels of mRNA were normalized by the reference genes *ACTIN 2/8*, *AT4G33380* and *SAND* and compared to +NC conditions which were to set to 1 in each experiment. Results present the average of ± SD of 3 replicates each in two independent experiments (-NC#1; -NC#2).

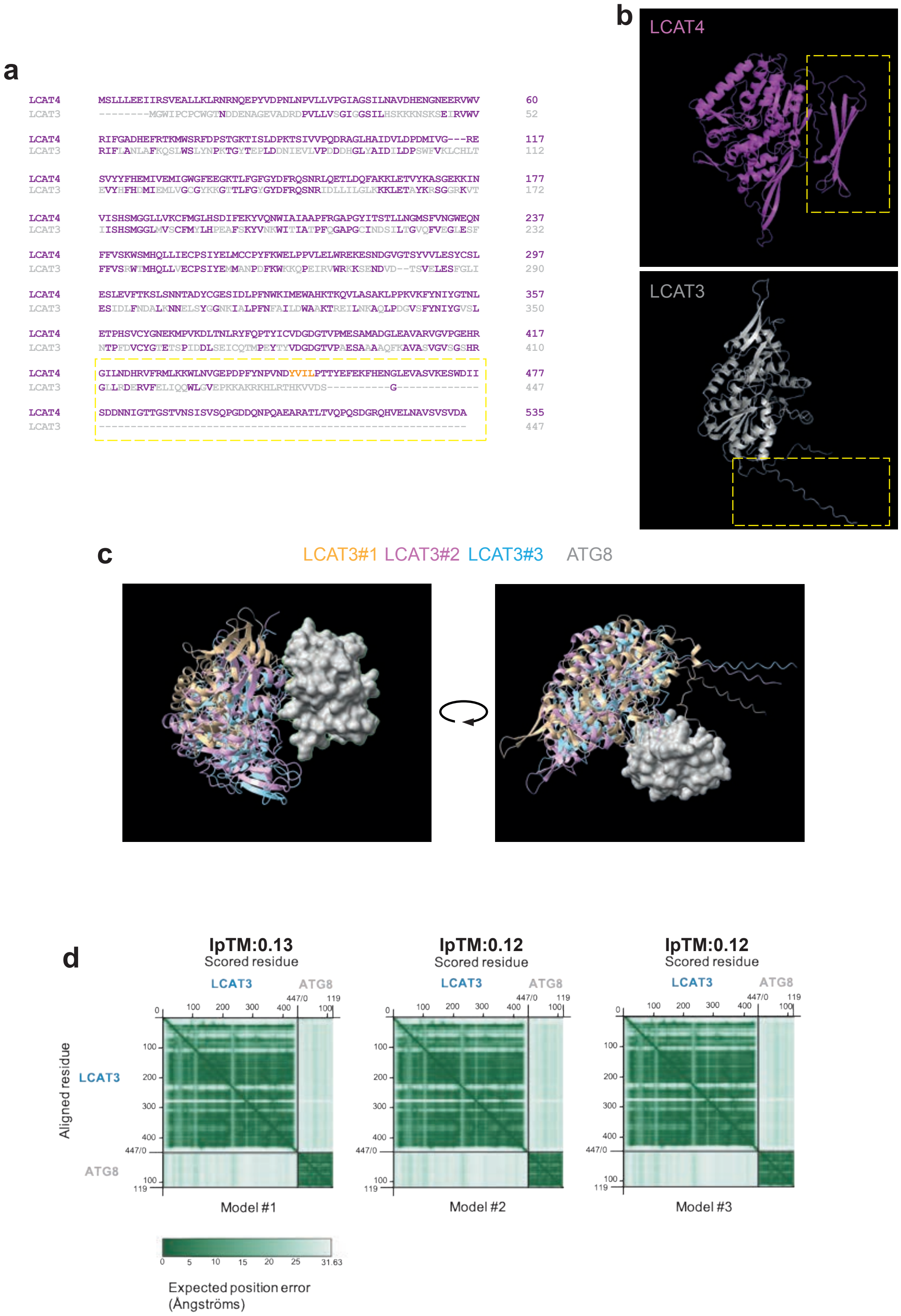

Supllementary Figure 6. Comparison of the protein and models of LCAT3 and LCAT4 suggest that LCAT3 does not interact with ATG8. **a** Alignment between the amnio acid sequence of LCAT4 (magenta) and LCAT3 (grey). The amino acids of LCAT3 identical to that of LCAT4 are indicated in magenta. The predicted AIM domain of LCAT4 is depicted in orange. The C-terminal tails of the protein are delineated by a yellow dotted square in (**a**) and (**b**). **b** AlphaFold3 models of LCAT3 and LCAT4 shows major differences in the C-terminal tail of the two proteins (yellow dotted rectangle). **c-d** Modelling the interface between LCAT3 and ATG8 did not result in a single orientation of ATG8 towards LCAT3 as shown for three best-scoring models calculated by AlphaFold3 multimer (**c**). Predicted aligned error (PAE) plots of three models of the LCAT3-ATG8 predict no interaction between the proteins (**d**).

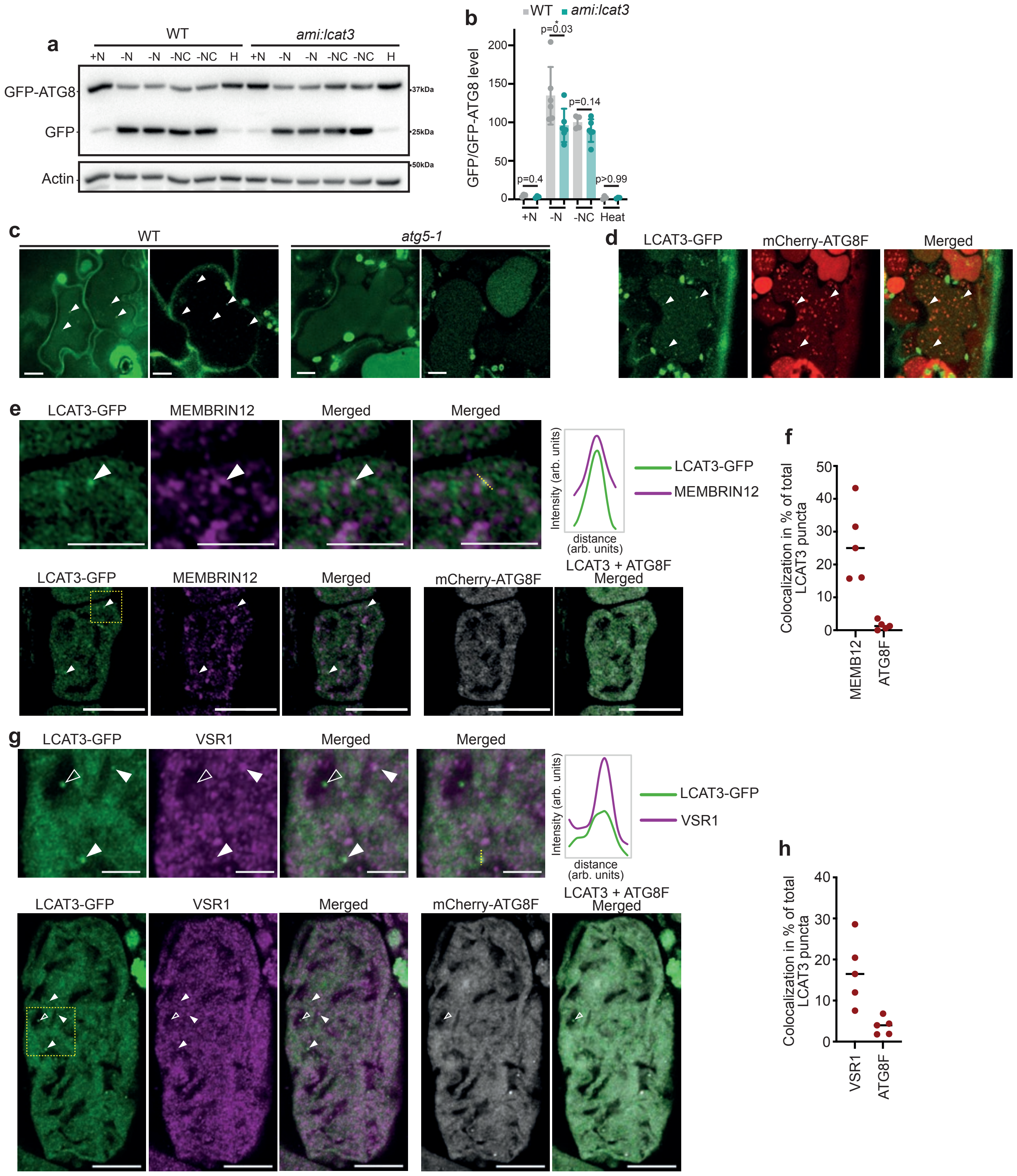

Supplementary Figure 7. Phenotype of LCAT3 knock down lines and subcellular localization of LCAT3

a Representative image of GFP-ATG8A processing assay in *amiRNA:LCAT3* compared to WT plants. 7-day-old seedlings were transferred to nutrient rich liquid medium (+N) supplemented with 10μM of β-estradiol for 16 hours to induce the expression of the amiRNA. Seedlings were then transferred to either nutrient rich liquid medium (+N), liquid medium depleted in nitrogen (-N) or depleted in nitrogen and carbon (-NC, in the dark) during 8h, all media containing 10 μM of β-estradiol. For heat stress (H), plants were transferred to +N liquid medium for 5 h at room temperature followed by 3 h at 37°C. The experiment was repeated three times with similar results. **b** Quantification of the ratio of GFP/GFP-ATG8A in (**a**) in percentage of the WT in -NC condition which was set to 1 in each experiment. Results are presented as the average ± SD with values of all replicates collected in three independent biological experiments; in +N, n=3, in -N and -NC, n=6, in H, n=3. Statistical differences were assessed using two-tailed Mann Whitney test compared to WT in the same condition (+N, -N, H) and two tailed one sample t.test compared to WT in the same condition (-NC); p values are indicated. **c-d** Autophagy is dispensable for the trafficking of LCAT3 into the vacuole. Similarly to mCherry-ATG8F, LCAT3-GFP shows a diffuse signal in the vacuole of WT or mCherry-ATG8F cotyledons as well as a dot-like localisation on autophagic bodies (**c**,**d** see co-localisation with mCherry-ATG8F-labeled autophagic bodies indicated by arrowheads). In cotyledons of the *atg5* mutant, LCAT3-GFP is also observed inside the vacuole but strictly as a diffuse signal (**c**). The experiment was repeated three times with similar results. Scale bar, 10 μm. **e-h** LCAT3 shows partial co-localization with markers of the secretory pathway. **e,g** Representative confocal images of root cells of plants co-expressing LCAT3-GFP and mCherry-ATG8F immunolabelled with antibodies against either MEMBRIN12 (MEMB12, **e**) or VSR1 (**g**) after 3 h of autophagy induction with concanamycin A (-NC+CA 1μM). Dotted squares in the bottom panels are enlarged in the top panel. Full arrowheads indicate co-localization between LCAT3 and MEMB12 or VSR1, empty arrowhead shows co-localization between LCAT3 ant ATG8 in the vacuole (**g**). Signal intensity profiles along the dotted lines are plotted in the boxed regions on the right and show co-localization between LCAT3 and MEMB12 or VSR1. Scale bar, 3 μm in top panels and 10 μm in bottom panels of each figure. The experiment was repeated in five independent roots with similar results. Quantification of (**e,g**) is provided in (**f,h**) and shows the percentage of LCAT3 puncta found colocalizing with MEMB12, VSR1 or ATG8F. Results show all individual values with median of n=5 independent roots within which a total of 592 (**f**) and 622 (**h**) LCAT3 puncta were analyzed.

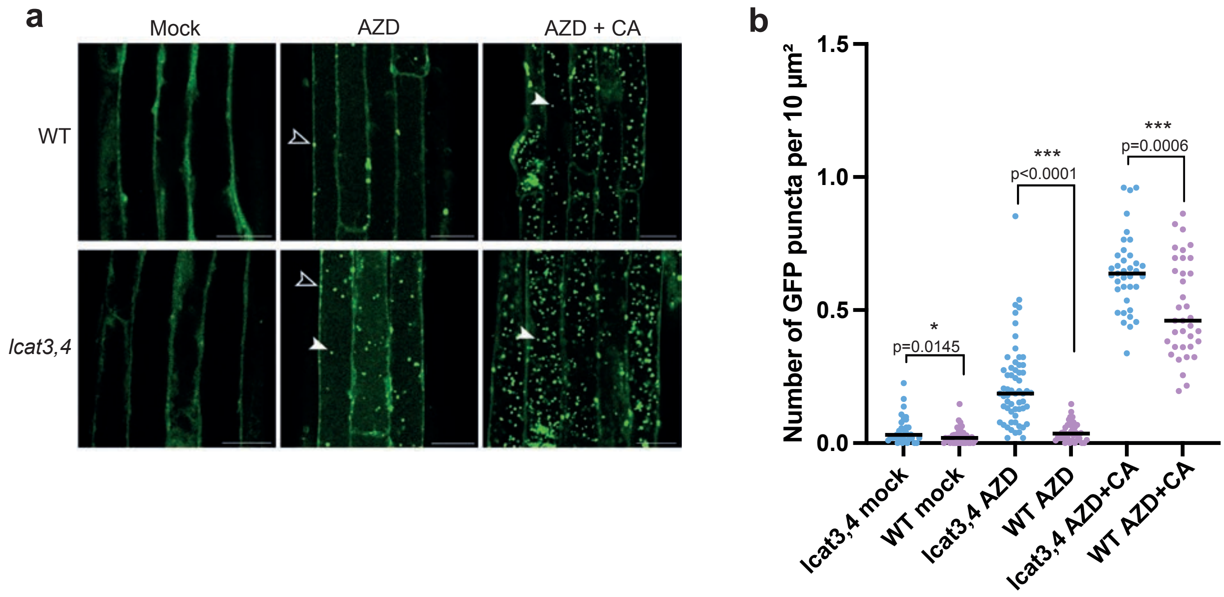

Supplementary Figure 8. Knocking out *LCAT3* and *LCAT4* results in the accumulation of autophagic bodies.
a Comparative confocal image analysis of 7-day-old seedling roots between *lcat3,4* line and wild-type (WT). Plants were transferred into 6-well plate with 3 ml of nutrient rich liquid complemented with nothing (Mock), AZD8055 (AZD, 5µM) or AZD + concanamycin A (AZD + CA; 1 µM). Filled arrow heads indicate the autophagic bodies and hollow arrow heads the autophagosomes. Scale bars, 20 µm. The experiment was repeated n times as indicated in (**b**), with similar results. **b** Quantification of GFP puncta in (**a**). Results are presented as the number of puncta per 10 µm² of root area and show the average and individual values, from left to right, n=36 images of 13 roots, n=38 images of 12 roots, n=59 images of 14 roots, n=36 of 11 roots, n=37 images of 12 roots and n=36 of 12 roots in 3 independent biological replicates. Statistical analyses were assessed using unpaired, two-tailed t-test.

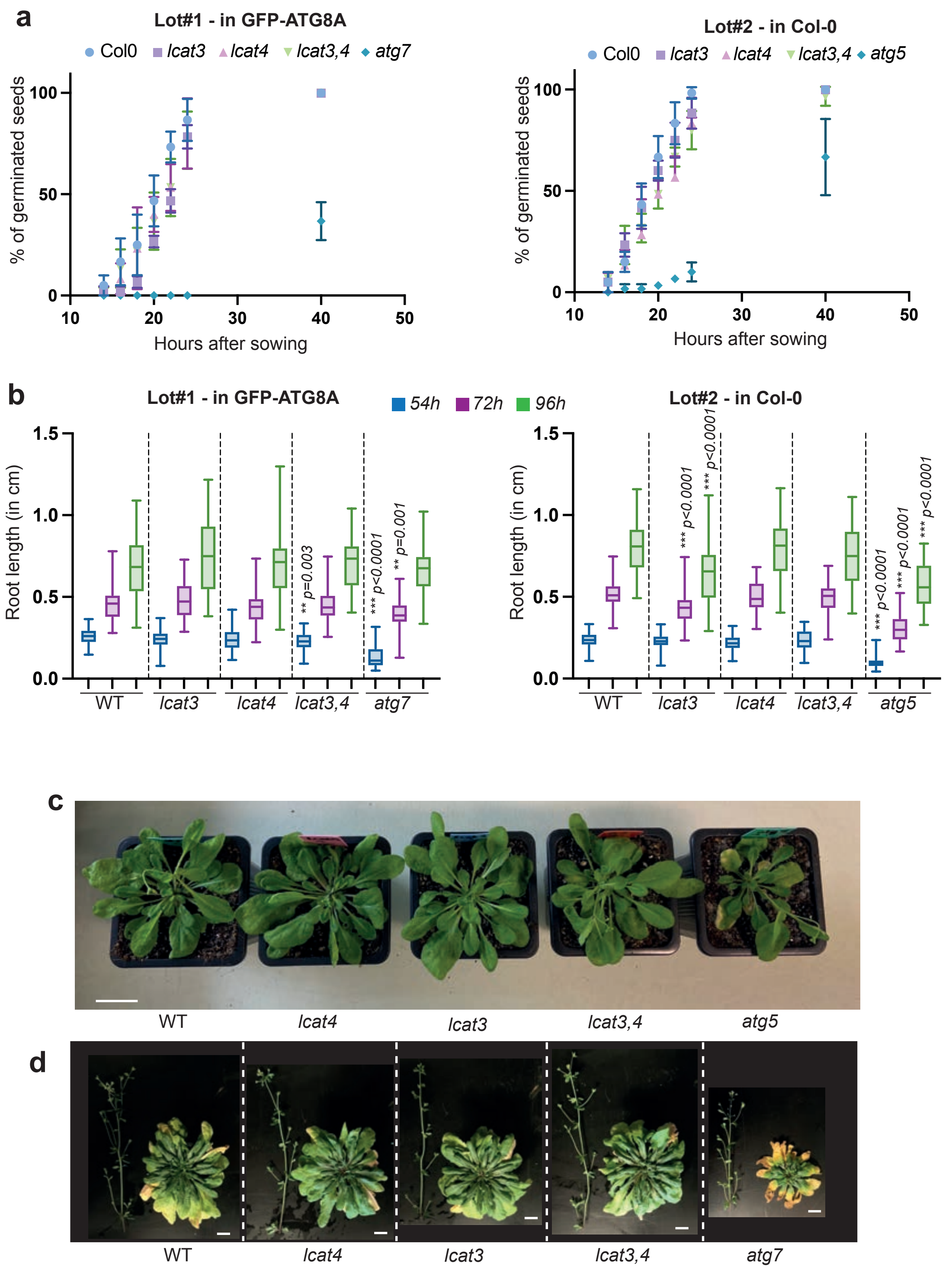

Supplementary Figure 9. Knock out mutants for *LCAT3*, *LCAT4* or both *LCAT3,4* show similar growth and development compared to WT plants.

a LCAT3 and LCAT4 are not required for proper seed germination while mutants with a complete block in the autophagy pathway (*atg5*, *atg7*) show germination impairments. 20-30 seeds per plate were sown on ½ MS + sucrose after two days of vernalization. Germination was assessed at the indicated times points. Results are expressed as percentage of germinated seeds relative to total seeds sown in 2-3 independent plates. Results of two biological experiments are presented (lot#1, lot#2). A total of 60 seeds were analyzed by independent experiment. **b** *LCAT3* and *LCAT4* knock out mutants show no to little changes in root elongation compared to WT plants and *atg* mutants. Roots were measured at the indicated times after seed sowing. Results are presented as box plots, the box extends from the 25th to 75th percentiles, the center line indicates the median, the whiskers show min and max values of 2-3 independent plates with a total of 60 seedlings per genotype per independent experiment. Results of two biological experiments are presented (lot#1, lot#2). Statistical differences were assessed using unpaired two-tailed t-test compared to WT at the same time after seed sowing. **c-d** Representative image of the vegetative growth and onset of senescence of *lcat3*, *lcat4* and *lcat3,4* mutants compared to WT plants and *atg* mutants. The experiment was repeated three times with similar results. Plants were grown on soil at 21°C under short-day conditions for 6 weeks (**c**) or 12 weeks (**d**). Scale bar: 1 cm.

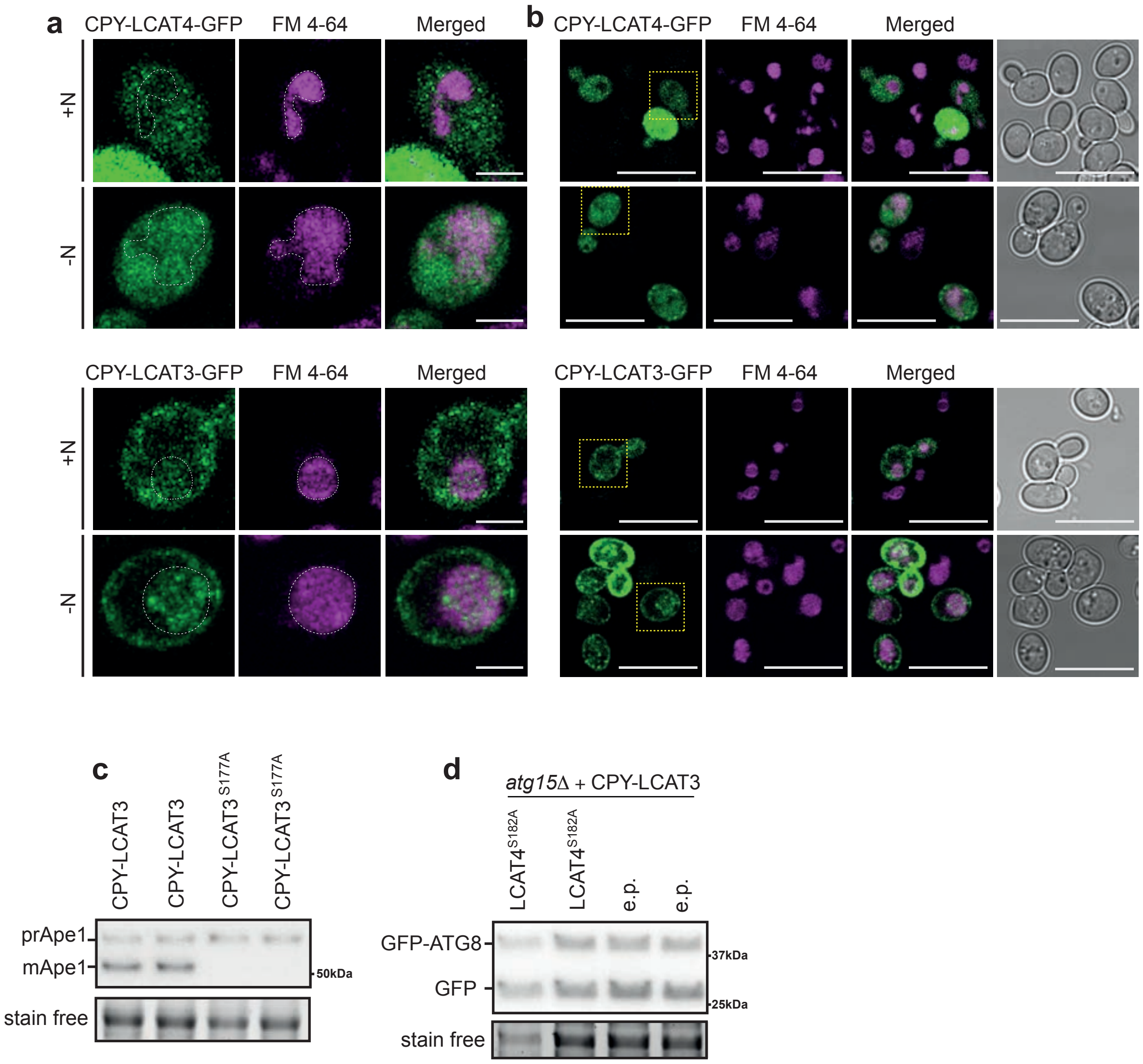

Supplementary Figure 10. Localization of CPY-LCAT3-GFP and CPY-LCAT4-GFP in *atg15Δ* yeast cells and autophagy assays using catalytic mutants of LCAT3 and LCAT4.
a-b CPY-LCAT3-GFP and CPY-LCAT4-GFP show signal in the vacuole of *atg15Δ* yeast cells. Cells were grown overnight in selective medium, diluted and grown until mid-log-phase in YPGal, stained with FM 4-64 (for 20 minutes) and chased in YP-Gal for 2 hours to label the vacuole (+N) prior to autophagy induction in SGal-N for 10h (-N). The experiment was repeated three times with similar results. (**a**), enlargement of (**b**), (dotted rectangle). Scale bar, 2 µm in (**a**) and 10 µm in (**b**). **c** Immunoblot analyses of Ape1 in *atg15Δ* cells transformed with either CPY-LCAT3 or CPY-LCAT3^{S177A} performed as in **Fig. 6a**. The experiment was repeated two times with similar results, results of 2 independent replicates are shown. **d** Immunoblot analyses of GFP-AtATG8A processing assays, performed in -N conditions as in **Fig. 6d**, in *atg15Δ* yeast co-transformed with CPY-LCAT3 and either the empty plasmid (e.p.) or LCAT4^{S182A}. The experiment was repeated two times with similar results, results of 2 independent replicates are shown.
