# Supplementary material for: A dual phospholipase system instructs membrane hydrolysis during the final stages of plant autophagy
Source: Nat Commun. 2026 May 14;17:6444. doi: 10.1038/s41467-026-73116-x (PMC13377104; doi:10.1038/s41467-026-73116-x)
Supplement: Supplementary file 2 — Descriptions of Additional Supplementary Files [file 41467_2026_73116_MOESM2_ESM.pdf]

Description of Additional Supplementary Files

Supplementary Data 1. Primers used for this study

Supplementary Data 2. Yeast strains used in this study

Supplementary Movie 1. LCAT3 can transiently associate with autophagosomes close to the vacuole. Movie representing LCAT3-GFP puncta associating with an autophagosome labeled with mCherry-ATG8F in the cytosol, at the edge of the vacuole. 7-dayold seedlings expressing both transgenes as in Fig. 4 were imaged by confocal microscopy after 3 hours of nutrient starvation with the addition of concanamycin A (-NC 3h+CA, 1 μM). Scale bar, 2 μm.
