# Supplementary material for: A dual phospholipase system instructs membrane hydrolysis during the final stages of plant autophagy
Source: Nat Commun. 2026 May 14;17:6444. doi: 10.1038/s41467-026-73116-x (PMC13377104; doi:10.1038/s41467-026-73116-x)
Supplement: Supplementary file 6 — Reporting Summary [file 41467_2026_73116_MOESM6_ESM.pdf]

nature portfolio

Corresponding author(s): Amélie Bernard
Last updated by author(s): Mar 3, 2026

Reporting Summary

Nature Portfolio wishes to improve the reproducibility of the work that we publish. This form provides structure for consistency and transparency in reporting. For further information on Nature Portfolio policies, see our Editorial Policies and the Editorial Policy Checklist.

Statistics

For all statistical analyses, confirm that the following items are present in the figure legend, table legend, main text, or Methods section.

n/a	Confirmed	
☐	☒	The exact sample size (*n*) for each experimental group/condition, given as a discrete number and unit of measurement
☐	☒	A statement on whether measurements were taken from distinct samples or whether the same sample was measured repeatedly
☐	☒	The statistical test(s) used AND whether they are one- or two-sided<br>*Only common tests should be described solely by name; describe more complex techniques in the Methods section.*
☒	☐	A description of all covariates tested
☒	☐	A description of any assumptions or corrections, such as tests of normality and adjustment for multiple comparisons
☐	☒	A full description of the statistical parameters including central tendency (e.g. means) or other basic estimates (e.g. regression coefficient) AND variation (e.g. standard deviation) or associated estimates of uncertainty (e.g. confidence intervals)
☐	☒	For null hypothesis testing, the test statistic (e.g. *F*, *t*, *r*) with confidence intervals, effect sizes, degrees of freedom and *P* value noted<br>*Give P values as exact values whenever suitable.*
☒	☐	For Bayesian analysis, information on the choice of priors and Markov chain Monte Carlo settings
☒	☐	For hierarchical and complex designs, identification of the appropriate level for tests and full reporting of outcomes
☒	☐	Estimates of effect sizes (e.g. Cohen's *d*, Pearson's *r*), indicating how they were calculated

Our web collection on statistics for biologists contains articles on many of the points above.

Software and code

Policy information about availability of computer code

Data collection	n/a
Data analysis	n/a

For manuscripts utilizing custom algorithms or software that are central to the research but not yet described in published literature, software must be made available to editors and reviewers. We strongly encourage code deposition in a community repository (e.g. GitHub). See the Nature Portfolio guidelines for submitting code & software for further information.

Data

Policy information about availability of data

All manuscripts must include a data availability statement. This statement should provide the following information, where applicable:

- Accession codes, unique identifiers, or web links for publicly available datasets
- A description of any restrictions on data availability
- For clinical datasets or third party data, please ensure that the statement adheres to our policy

Source data are provided with this paper

Research involving human participants, their data, or biological material

Policy information about studies with human participants or human data. See also policy information about sex, gender (identity/presentation), and sexual orientation and race, ethnicity and racism.

Reporting on sex and gender	n/a
Reporting on race, ethnicity, or other socially relevant groupings	n/a
Population characteristics	n/a
Recruitment	n/a
Ethics oversight	n/a

Note that full information on the approval of the study protocol must also be provided in the manuscript.

Field-specific reporting

Please select the one below that is the best fit for your research. If you are not sure, read the appropriate sections before making your selection.

☒ Life sciences ☐ Behavioural & social sciences ☐ Ecological, evolutionary & environmental sciences

For a reference copy of the document with all sections, see nature.com/documents/nr-reporting-summary-flat.pdf

Life sciences study design

All studies must disclose on these points even when the disclosure is negative.

Sample size	No sample size calculation was performed. Experiments were repeated in biological independent experiments as standard in the field. Sample size was chosen depending on the type of experiments (large number of replicates in quantitative microscopy analyses and survival analyses; moderate number of replicates for western blot analyses). Statistical analyses were performed to detect significant differences. In case where minor differences were observed, the sample size was increased to ensure that difference was significant and biologically relevant.
Data exclusions	No data were excluded
Replication	All experiments were performed in, at least, 3 independent replicates expect otherwise indicated in the figures. All attemps at replication were included in the data set. Statistical analyses indicate that differences are significant.
Randomization	Not relevant. Samples were not allocated into experimental groups. All samples were analyzed independently in each experiments.
Blinding	Blinding was considered whenever possible to keep track of experimental material.

Reporting for specific materials, systems and methods

We require information from authors about some types of materials, experimental systems and methods used in many studies. Here, indicate whether each material, system or method listed is relevant to your study. If you are not sure if a list item applies to your research, read the appropriate section before selecting a response.

Materials & experimental systems

n/a	Involved in the study	
☐	☒	Antibodies
☒	☐	Eukaryotic cell lines
☒	☐	Palaeontology and archaeology
☒	☐	Animals and other organisms
☒	☐	Clinical data
☒	☐	Dual use research of concern
☐	☒	Plants

Methods

n/a	Involved in the study	
☒	☐	ChIP-seq
☒	☐	Flow cytometry
☒	☐	MRI-based neuroimaging

Antibodies

Antibodies used	anti-MBP (Sigma Aldrich, # M1321), Anti-Mouse IgG-HRP Conjugate (Bio-Rad, #1706516), Anti-GST HRP-Conjugate (GE Healthcare, #

Antibodies used	RPN1236), anti-GFP (Roche, 11814460001); anti-RFP (Agrisera, AS153028); anti-MEMB12; anti-VSR1 (AGRISERA, AS20 4407), Atto 647N-coupled goat anti-rabbit IgG (Merck, 40839), anti-GFP (Torrey pines TP-401), goat anti-rabbit antibodies (Aurion GAR EM10), Goat anti-Rabbit secondary antibody (Biorad, #1706515), anti-Ape1
Validation	All antibodies were used as previously described (references are provided in the paper) and according to the manufacturer's instructions.

Dual use research of concern

Policy information about dual use research of concern

Hazards

Could the accidental, deliberate or reckless misuse of agents or technologies generated in the work, or the application of information presented in the manuscript, pose a threat to:

No	Yes	
☒	☐	Public health
☒	☐	National security
☒	☐	Crops and/or livestock
☒	☐	Ecosystems
☒	☐	Any other significant area

Experiments of concern

Does the work involve any of these experiments of concern:

No	Yes	
☒	☐	Demonstrate how to render a vaccine ineffective
☒	☐	Confer resistance to therapeutically useful antibiotics or antiviral agents
☒	☐	Enhance the virulence of a pathogen or render a nonpathogen virulent
☒	☐	Increase transmissibility of a pathogen
☒	☐	Alter the host range of a pathogen
☒	☐	Enable evasion of diagnostic/detection modalities
☒	☐	Enable the weaponization of a biological agent or toxin
☒	☐	Any other potentially harmful combination of experiments and agents

Plants

Seed stocks	Transgenic lines were used as previously described with references provided in the paper. TDNA insertion lines in LCAT3 (lcat3; SALK_035317) and LCAT4 (lcat4; SALK_147672.52.80) were obtained from the Nottingham Arabidopsis Stock Centre.
Novel plant genotypes	New transgenic lines were obtained by floral dip using agrobacterium tumefaciens introgression of transgenes or by crossing existing transgenic lines. Two to three independent homozygous and monoinsertional lines were produced and tested upon which one was selected for full analyses. All experiments were performed on T3 lines expect Fig.5i-o which was performed on T2 lines.
Authentication	Homozygous lines containing single construct insertion were selected based on Hygromycin B resistance, on red seed coat under epifluorescent microscopy and/or based on PCR (for T-DNA mutants and crosses with T-DNA mutants) as described in the paper.
