# Supplementary material for: A dual phospholipase system instructs membrane hydrolysis during the final stages of plant autophagy
Source: Nat Commun. 2026 May 14;17:6444. doi: 10.1038/s41467-026-73116-x (PMC13377104; doi:10.1038/s41467-026-73116-x)
Supplement: Supplementary file 7 — Transparent Peer Review file [file 41467_2026_73116_MOESM7_ESM.pdf]

nature portfolio

Peer Review File

A dual phospholipase system instructs membrane hydrolysis during the final stages of plant autophagy

Corresponding Author: Professor Amélie Bernard

This file contains all reviewer reports in order by version, followed by all author rebuttals in order by version.

Version 0:

Reviewer comments:

Reviewer #1

(Remarks to the Author)
Castets et al. report the roles of two Arabidopsis phospholipases LCAT3 and LCAT4 during the vacuolar stage of autophagy, proposing that they synergistically mediate autophagic body membrane degradation and drawing parallels to yeast Atg15. The study employs genetics, confocal imaging, protein-protein interaction assays, and heterologous yeast expression. While the work is novel and addresses an underexplored stage of plant autophagy, several conclusions are overstated, and the supporting evidence is insufficient in some cases. Additional validation or more cautious interpretation is required. I suppose that a journal more specialized for autophagy could be a suitable venue for the manuscript.
Major points
1. Subcellular localization of LCAT3 and LCAT4 mainly rely on pharmacological inhibition and fluorescence microscopy, without direct evidence from subcellular fractionation or immuno-EM.
2. LCAT4-ATG8 interaction support is limited to in vitro pull-down assays. In planta functional validation (e.g., Co-IP assay or AIM mutant rescue) is missing.
3. The claim that LCAT3 follows the classical ER-Golgi/TGN-MVB pathway relies mainly on concanamycin A treatment and lacks direct tracking evidence.
4. The lcat3/4 double mutant shows around 50% reduction in GFP flux assay while single mutants display no major defects. Also, lcat3/4 double mutant shows similar growth and development compared to WT plants. Describing them as “core components” is overstated.
5. Do LCAT3/4 specifically target autophagic body phospholipids rather than the tonoplast? Have lipidomics or liposome assays been conducted?
6. While previous reports suggest acidic activation of LCAT3/4, the current study lacks systematic validation (e.g., in vitro enzyme activity across pH gradients).
Minor points
1. Atg15 is a phospholipase B, while LCAT3/4 are PLA1/PLA2. The claim that their combination is “equivalent to PLB” lacks biochemical validation. This should be rephrased more cautiously as a “possible functional analogy.
2. In atg15Δ yeast, only CPY-targeted LCAT3 partially rescued autophagy, while LCAT4 did not. This highlights the importance of localization, yet the manuscript overinterprets this as functional equivalence.

Reviewer #2

(Remarks to the Author)
The manuscript by Castets et al. identifies two phospholipases as core components of the autophagy pathway. LCAT3 and LCAT4 are shown to be essential for autophagy, as plants deficient in these enzymes display impaired autophagic activity and increased sensitivity to nutrient starvation. The authors further demonstrate that the phospholipase activity of LCAT3 is required for the hydrolysis of autophagic membranes, a process that is enhanced by LCAT4.

Overall, this is a very exciting, elegant, and comprehensive study that identifies new components of the autophagy pathway and provides valuable insights into an understudied aspect of plant autophagy—namely, the processes occurring in the vacuole. I have only a few minor comments.

Figure 1: Is it possible to perform the same experiment with an AIM mutant of LCAT4 (at least in vitro). Does it still co-localize

with ATG8 and get delivered to the vacuole? However, it is also clear to me that sometimes mutating a single AIM is not sufficient since ATG8-interacting proteins may have also additional (unconventional) AIMs.

Figure 2: In panel (e), do the authors observe free RFP from LCAT4, which would indicate that it is eventually degraded? Does LCAT4 accumulate in atg5?

Figure 4: Although the expression profile of LCAT1 differs from LCAT3 and LCAT4, the authors could still examine its localization under autophagy-inducing conditions, as gene expression profiles do not necessarily predict function.

The discussion is very strong overall, but somewhat too long—I would consider condensing it for clarity. It would also help to provide a schematic model of the findings.

Reviewer #3

(Remarks to the Author)
This manuscript by Buridan et al. addresses the role of two phospholipases, LCAT3 and LCAT4, in the vacuolar degradation of autophagic bodies in plants. The authors provide compelling evidence that these enzymes act at a late step of the autophagy pathway and propose a model in which LCAT3 and LCAT4 act synergistically to disrupt autophagic body membranes. The study is timely, original, and of broad interest, particularly as it sheds light on a poorly characterized step of autophagy in plants.
While the overall story is convincing and the manuscript is clearly written, several points require additional clarification or experimental support before publication. I outline below a set of major and minor comments. Some of the experimental suggestions may be technically demanding, but even partial or alternative approaches, or at least further discussion in the text, would significantly strengthen the manuscript.

Specific point:
1) The authors propose a model in which LCAT3 localizes to the outer leaflet and LCAT4 to the inner leaflet of autophagic body membranes. While the indirect evidence is compelling, more direct localization data (e.g. immuno-EM, super-resolution imaging, or protease protection assays) would greatly strengthen this conclusion. I leave it to the authors to judge feasibility in plants, but even heterologous expression in yeast — where the authors already employed functional assays — could provide supportive evidence.

2) In Figure 1, the authors identify putative AIM motifs in LCAT4. Mutating the candidate AIM motifs and testing ATG8 binding would directly reveal which motif is functional. In addition, examining the subcellular co-localization of AIM-mutant LCAT4 with ATG8 would provide valuable evidence for the importance of the AIM in recruiting LCAT4 to autophagy compartments.

3) In Figure 4, the authors conclude that LCAT3 reaches the vacuole independently of autophagy. To strengthen this claim, it would be useful to examine whether LCAT3 still localizes to the vacuole in an *atg5* mutant background.

4) In Figure 5, the authors indicate that LCAT3 and LCAT4 are important for the degradation of autophagic bodies. To establish whether their phospholipase activities are indeed required, it would be valuable to test whether reintroduction of LCAT3 and/or LCAT4 into the *lcat3,4* double mutant rescues autophagy activity, and whether such rescue depends on their catalytic activity.

Minor comments:
1) In Extended data Figure 10, the fluorescence microscopy of CPY-LCAT3-GFP and CPY-LCAT4-GFP in yeast cells lacks a vacuolar marker. Including a vacuolar marker, or alternatively comparing with non-CPY tagged versions, would help clarify their vacuolar localization.

2) A schematic diagram illustrating the proposed localization of LCAT3 and LCAT4 within the vacuole would greatly aid in understanding the model.

3) Movie 1 appears to be missing.

4) The methodology for ATG8A compartment immune-isolation (Figure 1a) is not described in sufficient detail in the Material and methods section.

5) In Figure1b and 2a, it would be helpful if the figure legends specified what the arrowheads indicate.

Version 1:

Reviewer comments:

Reviewer #2

(Remarks to the Author)
The authors have addressed all of my comments satisfactorily. Congratulations on this excellent manuscript.

Reviewer #3

(Remarks to the Author)
The authors have sufficiently addressed my suggestions. I have no major further comments. However, in Figs. 3 and 4 and Extended data Fig. 8, the p-values are written using a comma instead of a decimal point. Please correct this to the standard format using a decimal point.

Reviewer #4

(Remarks to the Author)
I co-reviewed this manuscript with one of the reviewers who provided the listed reports. This is part of the Nature Communications initiative to facilitate training in peer review and to provide appropriate recognition for Early Career Researchers who co-review manuscripts.

Open Access This Peer Review File is licensed under a Creative Commons Attribution 4.0 International License, which permits use, sharing, adaptation, distribution and reproduction in any medium or format, as long as you give appropriate credit to the original author(s) and the source, provide a link to the Creative Commons license, and indicate if changes were made.
In cases where reviewers are anonymous, credit should be given to 'Anonymous Referee' and the source.
The images or other third party material in this Peer Review File are included in the article's Creative Commons license, unless indicated otherwise in a credit line to the material. If material is not included in the article's Creative Commons license and your intended use is not permitted by statutory regulation or exceeds the permitted use, you will need to obtain permission directly from the copyright holder.
To view a copy of this license, visit https://creativecommons.org/licenses/by/4.0/

Dear reviewers,

Thank you for your comments and suggestions according to which we performed additional experiments and modified our manuscript. We believe that these implementations strengthen our conclusions and the overall clarity of our main findings for which we now provide a schematic model (**Fig. 6g**).

Importantly, we noted that all three reviewers commented about the lack of *in planta* validation of the LCAT4-ATG8 interaction. We now provide results in **Fig. 5** showing that:

1) LCAT4 co-immunoprecipitates with ATG8A in Arabidopsis and, that the interaction is reduced by about 70% when the AIM domain of LCAT4 is mutated (LCAT4^{AIM}; **Fig. 5i-k**)

2) Expressing LCAT4-RFP in *lcat3,4* complements its autophagy phenotype both in terms of autophagy flux and degradation of autophagic bodies. In addition, reduced LCAT4-ATG8 interaction using the LCAT4^{AIM}-RFP transgene prevents the LCAT4 from co-localizing with autophagosome, from trafficking within the vacuole and from complementing either autophagy flux or the accumulation of autophagic bodies observed in *lcat3,4* (**Fig. 5m-o**)

Together, these additional results confirm the LCAT4-ATG8A interaction *in planta* and provides functional assays demonstrating the relevance of this interaction for the localization of LCAT4 and its function in the degradation of autophagic bodies in the vacuole.

Another important point raised by the reviewers is about the trafficking and subcellular localization of LCAT3.

1) We now add in the manuscript that LCAT3 was detected within the vacuole sap in Arabidopsis by previous proteomic analyses (Ohnishi et al., 2018), supporting our conclusion that LCAT3 localizes in the vacuole.

2) We performed transmission electronic microscopy and immunogold labelling of LCAT3-GFP expressing plants and we observed LCAT3-GFP particles on the membrane of autophagic bodies in the vacuole (**Fig. 4e**).

3) We analyzed the localization of LCAT3 in WT plants compared to the autophagy defective mutant *atg5* by performing transient transformation of LCAT3-GFP in Arabidopsis protoplasts (**Fig. 4f, g**) and cotyledons (**Extended data Fig. 7c-d)**. In both approaches, LCAT3-GFP was observed within the vacuole in either WT or *atg5* cells, demonstrating that autophagosome formation is dispensable for the trafficking of LCAT3 to the vacuolar lumen. This, coupled to the absence of colocalization between LCAT3 and ATG8, suggests that LCAT3 is not transported inside autophagosome/autophagic bodies but rather associates with autophagic bodies from their outer leaflet in the vacuole.

4) How LCAT3 reaches the vacuolar lumen remains an important question that will require extensive work, including the timely generation of transgenic material. We believe that this question is out of the scope of the current study which already places LCAT3 inside the vacuole and at autophagic bodies. Yet, we performed initial immunofluorescent analyses to test the hypothesis that LCAT3 could follow the canonical ER-Golgi-TGN pathway towards the vacuole. In these experiments presented in **Extended data Fig. 7e-h**, we confirmed that the co-localization between LCAT3 and ATG8F is very low (less than 5% of total LCAT3 puncta); in contrast, we found that about 25% of LCAT3 puncta co-localize with the Golgi marker MEMB12 and about 15% co-localize with a marker of post-golgi

vesicles, VSR1. Although these data support that LCAT3 could traffic through the golgi-TGN-vacuole pathway, as mentioned in the discussion section, we believe that these analyses are not sufficient to support a firm conclusion that will await future dedicated studies.

According to the reviewer's remarks, we now provide additional experimental and supporting evidence of our main conclusions and/or modified the text for more cautious interpretations. We answered all questions and comments and performed most of the suggested experiments with a few exceptions; proper justification is provided in the **point-to-point responses below**. We hope that you agree with us that these additional experiments strengthen our main conclusions and that you will consider our manuscript for publication in Nature Communications.

Point to point responses:

Reviewer #1 (Remarks to the Author)

Castets et al. report the roles of two Arabidopsis phospholipases LCAT3 and LCAT4 during the vacuolar stage of autophagy, proposing that they synergistically mediate autophagic body membrane degradation and drawing parallels to yeast Atg15. The study employs genetics, confocal imaging, protein-protein interaction assays, and heterologous yeast expression. While the work is novel and addresses an underexplored stage of plant autophagy, several conclusions are overstated, and the supporting evidence is insufficient in some cases. Additional validation or more cautious interpretation is required. I suppose that a journal more specialized for autophagy could be a suitable venue for the manuscript.

We thank the reviewer for his/her comments and suggestions. We agree that considering LCAT3 and LCAT4 as core components of the autophagy machinery is overstated and we modified the text accordingly to state instead that 'LCAT3 and LCAT4 are components of the autophagy machinery' (see below). Additionally, we now provide data that strengthen our main conclusions, i.e. that LCAT4 interacts with ATG8 *in planta*, and that LCAT4 uses autophagy as a transport pathway to reach the vacuole in which it participates in the turn-over of autophagic bodies. In contrast, our additional experiments demonstrate that LCAT3 localizes in the vacuole, and that its trafficking is independent of autophagosome formation. In line with reviewer#2 and #3, we believe that the conclusions of our manuscript are original, exiting, of broad interest and shed light on a poorly described step of the autophagy pathway. We hope that the reviewer will share our interest and support the publication of our research in Nature Communications.

Major points

1. Subcellular localization of LCAT3 and LCAT4 mainly rely on pharmacological inhibition and fluorescence microscopy, without direct evidence from subcellular fractionation or immuno-EM.
2. LCAT4-ATG8 interaction support is limited to in vitro pull-down assays. In planta functional validation (e.g., Co-IP assay or AIM mutant rescue) is missing.

To answer points 1 and 2: We thank the reviewer for these helpful comments. Concerning the localization of LCAT4, we show the association of the protein with autophagic compartments by both subcellular fractionation (**Fig. 1a**) and fluorescent microscopy in multiple conditions including without pharmacological inhibition (**Fig. 1b**). We also show a direct interaction of LCAT4 with ATG8 *in vitro*

(**Fig. 1g-h; extended data Fig. 3a,b**) that we now confirm *in vivo* with coIP experiments presented in **Fig. 5i-k**. Our additional data further indicate that mutations in the AIM domain of LCAT4 cause a large decrease in ATG8 interaction *in vivo* and fails to complement the autophagy defects of the *lcat3,4* double mutant compared to wild-type LCAT4 (in terms of autophagy activity monitored by GFP-ATG8 assay and accumulation of autophagic bodies). Additionally, we found that $LCAT4^{AIM}$ is mostly localized in the cytosol compared to $LCAT4^{WT}$ which traffics in the vacuole upon autophagy induction. We believe that the additional experiments and results strengthen our conclusion that a direct interaction between LCAT4 and ATG8 mediates the recruitment of LCAT4 to autophagosomes/autophagic bodies for its trafficking in the vacuole upon autophagy induction. Furthermore, this interaction is important for autophagy since reducing it prevents LCAT4 to traffic to the vacuole and to hydrolyze the membrane of autophagic bodies (see schematic working model in **Fig. 6g**).

Regarding LCAT3, we acknowledge that most of our experiments are performed in the presence of concanamycin A. This is nevertheless an obligatory tool (1) to visualize GFP-tagged proteins in plant vacuoles because of the quenching of the GFP fluorescence in such acidic environment and (2) to accumulate autophagic bodies which are otherwise rapidly hydrolyzed. However, as now mentioned page 8, line 234, LCAT3 was detected in the vacuolar sap by previous proteomics analyses (Ohnishi et al., 2018), supporting our data and conclusion that LCAT3 localizes in the vacuolar lumen. Furthermore, according to the reviewer's suggestion, we performed TEM and immunogold labelling of LCAT3-GFP expressing plants showing LCAT3-GFP particles on the membrane of autophagic bodies in the vacuole (**Fig. 4e**). Finally, we now provide additional experiments demonstrating that LCAT3 traffics to the vacuole independently of autophagy and suggesting that it may traffic through the secretory pathway (see next comment).

3. The claim that LCAT3 follows the classical ER-Golgi/TGN-MVB pathway relies mainly on concanamycin A treatment and lacks direct tracking evidence.

We agree with the reviewer that the trafficking route for LCAT3, potentially following the classical ER-Golgi/TGN-MVB pathway, lacks direct tracking evidence, which is why we emphasized in the text that this is only an hypothesis (See main text of the previous version 'The absence of LCAT3 on autophagosomes coupled with the presence of LCAT3 in the vacuole suggests that, in contrast to LCAT4, LCAT3 traffics to the vacuole through the canonical pathway for vacuolar resident proteins (…); or 'Despite this trafficking route remaining to be characterized, our results clearly demonstrate that a portion of LCAT3 localizes in the vacuole lumen, likely independently of autophagy (…)).

We now provide additional data testing the localization of LCAT3 when transiently expressed in either protoplasts or cotyledons of Arabidopsis in autophagy-inducing conditions and in presence of concanamycin A. In WT cotyledons, we found LCAT3 in the vacuole, either in dot-like structures colocalizing with autophagic bodies or as a diffuse signal (mCherry-ATG8F showed a similar pattern either dotty or diffuse, **Extended data Fig. 7c-d**). In the *atg5* mutant, which cannot form autophagosomes or autophagic bodies, LCAT3-GFP was also observed inside the vacuole, strictly as a diffuse consistent with the absence of autophagic bodies (**Extended data Fig. 7c-d**). These results were recapitulated by transiently transforming LCAT3-GFP in Arabidopsis protoplasts showing, similarly, that LCAT3-GFP is found in the vacuolar lumen in WT cells and in *atg5* cells (**Fig. 4f, g**). These additional experiments demonstrate that autophagy is dispensable for the trafficking of LCAT3 to the vacuolar lumen.

We also performed colocalization assays using immunofluorescent microscopy on fixed root samples using a marker of the Golgi (MEMBRIN12) or a marker of post-Golgi vesicles (TGN/MVB, VSR1). Data presented in **Extended data Fig. 7e-h** confirmed that LCAT3 rarely co-localizes with ATG8 but

instead shows partial co-localization with MEMBRIN12 (25% of total LCAT3 puncta) and VSR1 (15% of total LCAT3 puncta) suggesting that at least a portion of LCAT3 traffics through the Golgi-TGN-vacuole in these conditions. While we consider that these experiments are not sufficient to fully address the trafficking route of LCAT3 -which will require further dedicated analyses out of the scope of this publication- we believe that they provide additional elements supporting the hypothesis that LCAT3 traffics to the vacuole largely independently of autophagy, and suggesting that it may traffic -at least partially- through the canonical 'Golgi-TGN-MVB' pathway.

4. The lcat3/4 double mutant shows around 50% reduction in GFP flux assay while single mutants display no major defects. Also, lcat3/4 double mutant shows similar growth and development compared to WT plants. Describing them as "core components" is overstated.

This is a good point and we thank the reviewer for it as we do not want to overstate any conclusions. We now changed the manuscript accordingly by proposing that 'LCAT3 and LCAT4 are components of the autophagy machinery' (lines 32-33) or 'part of the machinery of autophagy' (lines 450-451).

5. Do LCAT3/4 specifically target autophagic body phospholipids rather than the tonoplast? Have lipidomics or liposome assays been conducted?

This is a very interesting question that we hope to be able to address in the upcoming years. At this time, we have not performed liposome assays to test this hypothesis and we believe that these experiments are out of the scope of this initial study (for example, the comprehensive lipid composition of the Arabidopsis tonoplast has not even been characterized to date) but would represent an exciting perspective for future projects. For better clarity and to avoid overstatement or excessive speculation, we have modified the discussion section and now mention that 'Whether and how a specific localization of LCAT3 and/or LCAT4 at the membrane of autophagic bodies could spatially determine the site of their activity and thereby prevent tonoplast hydrolysis remains to be investigated.' Lines 517-519.

6. While previous reports suggest acidic activation of LCAT3/4, the current study lacks systematic validation (e.g., in vitro enzyme activity across pH gradients).

At this time, we have decided not to include systematic validation of previously published results, notably because we have no reason to question these data. We hope that the reviewer will agree with us that although it would be interesting to compare the activity and pH requirements of LCAT3 and LCAT4 in future investigations, this is not essential for the conclusion of our study, which shows that they both localize in the vacuole, converge on autophagic bodies and are required for their hydrolysis.

Minor points

1. Atg15 is a phospholipase B, while LCAT3/4 are PLA1/PLA2. The claim that their combination is "equivalent to PLB" lacks biochemical validation. This should be rephrased more cautiously as a "possible functional analogy.

This is a fair point and we changed the text accordingly to 'Whether and how the combined action of LCAT3 and LCAT4 could represent a functional analogy with the phospholipase B activity of Atg15 awaits future studies.' Lines 482-484.

2. In atg15Δ yeast, only CPY-targeted LCAT3 partially rescued autophagy, while LCAT4 did not. This

highlights the importance of localization, yet the manuscript overinterprets this as functional equivalence.

We are not sure to fully understand the reviewer's comment. As mentioned in the manuscript, when expressed alone, LCAT4 or CPY-LCAT4 did not rescue autophagy (page 13, **Fig.6c,e**) which indicate a certain specificity between LCAT3 or LCAT4 in the recognition of the autophagic body membrane when expressed in the vacuolar lumen. We now modified the discussion to mention that: 'the expression of LCAT3 in the vacuole of yeast cells can partially complement the absence of Atg15, suggesting that the two proteins may share some functional homologies' (line 479).

Reviewer #2 (Remarks to the Author):

The manuscript by Castets et al. identifies two phospholipases as core components of the autophagy pathway. LCAT3 and LCAT4 are shown to be essential for autophagy, as plants deficient in these enzymes display impaired autophagic activity and increased sensitivity to nutrient starvation. The authors further demonstrate that the phospholipase activity of LCAT3 is required for the hydrolysis of autophagic membranes, a process that is enhanced by LCAT4.

Overall, this is a very exciting, elegant, and comprehensive study that identifies new components of the autophagy pathway and provides valuable insights into an understudied aspect of plant autophagy—namely, the processes occurring in the vacuole. I have only a few minor comments.

We thank the reviewer for his/her very positive comments and suggestions.

Figure 1: Is it possible to perform the same experiment with an AIM mutant of LCAT4 (at least in vitro). Does it still co-localize with ATG8 and get delivered to the vacuole? However, it is also clear to me that sometimes mutating a single AIM is not sufficient since ATG8-interacting proteins may have also additional (unconventional) AIMs.

We thank the reviewer for this suggestion and we agree that it was a critical point to strengthen our conclusions. We therefore have updated the manuscript with a set of additional experiments showing LCAT4-ATG8 interaction *in vivo* using CoIP experiments (**Fig. 5i,k**). Our data further indicate that mutations in the AIM domain of LCAT4 causes a large decrease in ATG8 interaction *in vivo* and fails to complement the autophagy defects of the *lcat3,4* double mutant compared to wild-type LCAT4 (in terms of autophagy activity monitored by GFP-ATG8 assays and accumulation of autophagic bodies). Additionally, we found that $LCAT4^{AIM}$ is mostly localized in the cytosol compared to $LCAT4^{WT}$ which traffics in the vacuole upon autophagy induction. In sum, we believe that these additional results now demonstrate that a direct interaction between LCAT4 and ATG8 mediates the recruitment of LCAT4 to autophagosomes/autophagic bodies for its trafficking in the vacuole upon autophagy induction. In addition, reducing this interaction prevents LCAT4 to traffic to the vacuole and to hydrolyze the membrane of autophagic bodies (see schematic working model in **Fig. 6g**).

Figure 2: In panel (e), do the authors observe free RFP from LCAT4, which would indicate that it is eventually degraded? Does LCAT4 accumulate in atg5?

The additional experiments that we now provide in **Fig. 5i-j** show that the $LCAT4^{AIM}$ mutant significantly and largely over-accumulates compared to $LCAT4^{WT}$ indicating that preventing the

vacuolar trafficking of the protein leads to its stabilization and suggesting that it is eventually degraded in the vacuole. To test this hypothesis, we assessed the level of LCAT4-RFP and free RFP upon long-term starvation (8h) in the presence or absence of concanamycin A. Although we did not detect free RFP in either condition, we found that the level of LCAT4 decreases upon prolonged -NC conditions and that this is partially prevented by blocking autophagy degradation using concanamycin A (**Extended data Fig. 3d-e**). We therefore now mention this result in the text, stipulating that although LCAT4 is not initially degraded by autophagy, the protein eventually gets degraded upon prolonged autophagy induction and vacuolar trafficking (lines 188-190).

Figure 4: Although the expression profile of LCAT1 differs from LCAT3 and LCAT4, the authors could still examine its localization under autophagy-inducing conditions, as gene expression profiles do not necessarily predict function.
This is a fair point and we agree with the reviewer that it would be very interesting to examine the localization and function of LCAT1 in autophagy. We nevertheless believe that this falls out of the scope of the current paper and rather open perspectives for future studies. We therefore modified the text accordingly page 8, lines 254-256: 'Although we acknowledge that gene expression does not necessarily predict function, the repression of *LCAT1* led us to not consider this gene further in this study, leaving future investigations to explore whether it has a role in autophagy'.

The discussion is very strong overall, but somewhat too long—I would consider condensing it for clarity. It would also help to provide a schematic model of the findings.

We agree. The revised discussion is condensed as the reviewer suggested and it additionally now provides a model summarizing our major findings for better clarity (**Fig. 6g)**.

Reviewer #3 (Remarks to the Author):

This manuscript by Buridan et al. addresses the role of two phospholipases, LCAT3 and LCAT4, in the vacuolar degradation of autophagic bodies in plants. The authors provide compelling evidence that these enzymes act at a late step of the autophagy pathway and propose a model in which LCAT3 and LCAT4 act synergistically to disrupt autophagic body membranes. The study is timely, original, and of broad interest, particularly as it sheds light on a poorly characterized step of autophagy in plants. While the overall story is convincing and the manuscript is clearly written, several points require additional clarification or experimental support before publication. I outline below a set of major and minor comments. Some of the experimental suggestions may be technically demanding, but even partial or alternative approaches, or at least further discussion in the text, would significantly strengthen the manuscript.
We thank the reviewer for his/her helpful comments and suggestions. Because of the time required to generate transgenic plants (about 1.5 years), we were unable to perform some of the additional experiments proposed by the reviewer. Alternatively, when possible, we performed transient assays to start addressing his/her comments and modified the text to provide additional discussion for better clarity.

Specific point:

1) The authors propose a model in which LCAT3 localizes to the outer leaflet and LCAT4 to the inner leaflet of autophagic body membranes. While the indirect evidence is compelling, more direct localization data (e.g. immuno-EM, super-resolution imaging, or protease protection assays) would greatly strengthen this conclusion. I leave it to the authors to judge feasibility in plants, but even heterologous expression in yeast — where the authors already employed functional assays — could provide supportive evidence.

We thank the reviewer for this comment and agree that it would be of great interest to be able to discriminate inner vs outer membrane localization. However, at this time, we consider that the imaging techniques having the required resolution to address this point are limited in plants. We think that immuno-gold labelling would not be appropriate as the length of an IgG protein is more than double the width of a phospholipid bilayer, so detection of a nano gold on one side of a membrane would not necessarily guarantee that the epitope is also on the same side. Super-resolution imaging could be an option but it is only starting to emerge in plants and would require the lengthy generation of novel transgenic material with no guarantee of success.

We therefore instead tested protease protection assays on microsomal fraction containing autophagic vesicles as previously published (Zhao et al., 2022). Representative images of these experiments are provided below in **Additional Fig.1**. In these conditions we found that, similar to the autophagy-adaptor NBR1 or to GFP-ATG8A, LCAT4 is partially protected from proteinase K, suggesting that LCAT4 is found inside membrane vesicles. Given that LCAT4 shows a 70% co-localization with autophagic compartments (autophagosomes, autophagic bodies), this suggest that LCAT4 is found inside autophagic vesicles. This is further supported by our complementation assays of the *lcat3,4* KO with an $LCAT4^{AIM}$ mutant vs $LCAT4^{WT}$. We now show in **Fig. 5i-l** that mutations in the AIM domain largely reduce LCAT4-ATG8 interaction *in vivo* which leads to an accumulation of the protein in the cytosol when $LCAT4^{WT}$ traffics to the vacuolar lumen. Consequently, $LCAT4^{AIM}$ fails to complement the autophagy phenotype of *lcat3,4* in terms of both autophagy flux and accumulation of autophagic bodies (**Fig. 5i-o**). Together, we believe that these additional experiments support that LCAT4 is found inside autophagosome/autophagic bodies through a direct ATG8 interaction.

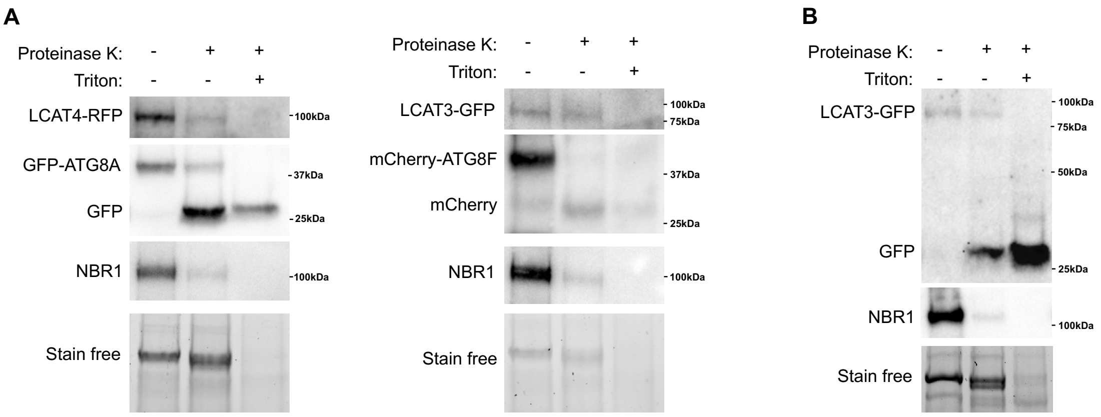

Additional Figure 1. Western blot analyses of protease protection assays perfomed as in Zhao et al., 2022 with 7-day old seedlings treated for 3 h with AZD + CA (**A**, representative image of n=3 for LCAT4, n=1 for LCAT3) or 3 h in –NC + CA (**B**, representative image of n=3).

We also tested protease protection assays with LCAT3-GFP (see **Additional Fig. 1A, B**) and found that, similar to LCAT4, NBR1 and ATG8, LCAT3 is also partially protected from proteinase K treatment, showing that LCAT3 is partially within membrane vesicles. However, given that LCAT3 largely accumulates in dot-like structures scattered in the cytoplasm which do not co-localize with autophagosomes in these conditions (**Fig. 4b; Fig. 4f**, middle panel), we cannot conclude that LCAT3 is found inside autophagic compartments but rather than LCAT3 is found inside unidentified membrane vesicles. Additional immunofluorescent experiments presented in **Extended data Fig. 7e-h** suggest that at least a portion of such vesicles are related to the secretory pathway as LCAT3 was found partially co-localizing with markers of Golgi or post-Golgi compartments (see below).

To avoid any confusion, we decided not to include these protease protection assay analyses in the manuscript, which we consider to be inconclusive in determining whether LCAT3 is found inside autophagic bodies or not. Instead, we now provide additional analyses showing that LCAT3 is detected within the vacuole of the *atg5* mutant (see below, **Fig. 4f-g** and **Extended data Fig. 7c-d**), indicating that the protein can traffic to the vacuole independently of autophagosome formation. We believe that these data strengthen our conclusion that LCAT3 is not transported/found inside autophagic bodies but rather associates with autophagic bodies once in the vacuole, hence likely recognizing their outer leaflet. However, we acknowledge that this does not provide direct evidence of such localization and we therefore modified the text to be more cautious with our proposed conclusion (page 13, lines 416-417; page 14, lines 458-459).

2) In Figure 1, the authors identify putative AIM motifs in LCAT4. Mutating the candidate AIM motifs and testing ATG8 binding would directly reveal which motif is functional. In addition, examining the subcellular co-localization of AIM-mutant LCAT4 with ATG8 would provide valuable evidence for the importance of the AIM in recruiting LCAT4 to autophagy compartments.

We agree with the reviewer that this is a valuable piece of information missing in our previous manuscript. We indeed already identified that important point and had been working on generating the required transgenics prior to the first submission. As mentioned in the previous comment, we now provide a whole set of experiments in **Fig.5i-o** showing that:

- LCAT4 interacts with ATG8 *in vivo*, using coIP (**Fig. 5i,k**)
- Mutating the predicted AIM domain of LCAT4 largely reduces this interaction (**Fig. 5i-k**)
- Preventing LCAT4-ATG8 interaction prevents the vacuolar trafficking of LCAT4 as shown by the localization of $LCAT4^{AIM}$ in the cytosol when $LCAT4^{WT}$ is mostly found in the vacuolar lumen upon autophagy induction (**Fig. 5m,n**)
- Expressing $LCAT4^{AIM}$ in the double *lcat3,4* mutant fails to restore autophagy flux (**Fig. 5i,l**) and hydrolysis of autophagic bodies compared to $LCAT4^{WT}$ (**Fig.5o**)

This set of experiments strengthen the importance of the LCAT4-ATG8 interaction in the recruitment of LCAT4 to the autophagosome/autophagic body and further support that autophagy acts as a trafficking pathway to the vacuole for LCAT4 in these conditions.

3) In Figure 4, the authors conclude that LCAT3 reaches the vacuole independently of autophagy. To strengthen this claim, it would be useful to examine whether LCAT3 still localizes to the vacuole in an *atg5* mutant background.

We thank the reviewer for this helpful suggestion. To address this point, we used transient expression assays, as fast way of testing the subcellular localization of proteins of interest. LCAT3-GFP was transformed in cotyledons of WT seedlings, mCherry-ATG8F expressing seedlings or *atg5* seedlings

prior to autophagy induction + concanamycin A treatment to visualize autophagic bodies. In WT plants, we found LCAT3 in the vacuole, either in dot-like structures colocalizing with autophagic bodies or as a diffuse signal (mCherry-ATG8F showed a similar pattern either dotty or diffuse in the vacuole; **Extended data Fig.7c-d**). In the *atg5* mutant, which cannot form autophagosomes or autophagic bodies, we never observed LCAT3 on dot-like structures in the vacuole; instead, we observed a diffuse signal of the protein within the vacuolar lumen in multiple cells. These results were recapitulated by transiently transforming LCAT3-GFP in Arabidopsis protoplasts showing, similarly, that LCAT3-GFP is found in the vacuolar lumen in WT cells and in *atg5*cells (**Fig. 4f-g**). These additional experiments demonstrate that autophagy is dispensable for the trafficking of LCAT3 to the vacuolar lumen.

Additionally, we performed colocalization assays (presented in **Extended data Fig. 7 e-h**) using immunofluorescent experiments on fixed root samples using a marker of the Golgi (MEMBRIN12) or a marker of TGN/MVBs (VSR1). These additional data show that LCAT3 rarely co-localizes with ATG8 but instead shows substantial co-localization with MEMBRIN12 (25%) and VSR1 (15%) suggesting that at least a portion of LCAT3 may traffic through the Golgi-TGN-MVB in these conditions. While we consider that these experiments are not sufficient to fully address the trafficking route of LCAT3 -which will require further dedicated analyses out of the scope of this publication- we believe that they provide additional elements demonstrating that LCAT3 traffics to the vacuole largely independently of autophagy, and suggesting that LCAT3 may at least be partially transported through the canonical 'Golgi-TGN-MVB' pathway.

4) In Figure 5, the authors indicate that LCAT3 and LCAT4 are important for the degradation of autophagic bodies. To establish whether their phospholipase activities are indeed required, it would be valuable to test whether reintroduction of LCAT3 and/or LCAT4 into the *lcat3,4* double mutant rescues autophagy activity, and whether such rescue depends on their catalytic activity.

We thank the reviewer for this suggestion; while we do agree that it would be valuable to perform these complementation assays we are also limited by time constraints as the generation of these transgenics lines would take up to 1.5 years prior to perform the experiments. Using transgenics that we had already started to generate prior to the first submission, we now demonstrate in **Fig.5i-o** that complementing the *lcat3,4* double mutant with LCAT4 rescues defects in the hydrolysis of autophagic bodies and this is accompanied with a rescue of autophagic flux compared to *lcat3,4*. In addition, we believe that the yeast data shown in **Fig.6** already provide additional support to demonstrate the importance of the phospholipase activity of LCAT3 and LCAT4 in the hydrolysis of autophagic bodies (note that point mutation in the catalytic site of LCAT3 or LCAT4 were found to prevent the hydrolysis of autophagic bodies in these conditions as now shown in **Extended data Fig. 10c-d**). We hope that the reviewer agrees with us that these elements are sufficient to support our conclusion that the phospholipase activity of LCAT3 and LCAT4 participates in the membrane hydrolysis of autophagic bodies.

Minor comments:

1) In Extended data Figure 10, the fluorescence microscopy of CPY-LCAT3-GFP and CPY-LCAT4-GFP in yeast cells lacks a vacuolar marker. Including a vacuolar marker, or alternatively comparing with non-CPY tagged versions, would help clarify their vacuolar localization.

We now provide new data using FM 4-64 labeling of yeast vacuoles confirming that CPY-LCAT4-GFP or CPY-LCAT3-GFP are observed within the vacuole as presented in **Extended data Fig. 10a-b** in both +N and -N conditions. According to the protocol for FM 4-64 labelling, we performed these experiments in complete medium (YPGal) when our previous observations had been done in -T minimal medium.

We found that in YPGal, the signal of both proteins is observed within the vacuole (in addition to the cytosol) where they further accumulate in -N conditions.

2) A schematic diagram illustrating the proposed localization of LCAT3 and LCAT4 within the vacuole would greatly aid in understanding the model.

Thank you for this suggestion, we now provide a schematic model in **Fig. 6g**.

3) Movie 1 appears to be missing.
It was indeed, we apologize for that; movie 1 is now provided.

4) The methodology for ATG8A compartment immune-isolation (Figure 1a) is not described in sufficient detail in the Material and methods section.

We apologize for the lack of details concerning these experiments, reference to the appropriate study where immuno-isolations were performed is now provided in the updated versions of the manuscript (line 667).

5) In Figure1b and 2a, it would be helpful if the figure legends specified what the arrowheads indicate.
Thank you for pointing that out, we now provide the information in the legends of **Fig. 1** and **Fig. 2**.

Reviewer #2 (Remarks to the Author):

The authors have addressed all of my comments satisfactorily. Congratulations on this excellent manuscript.

We thank the reviewer for her/his remarks and for helping us putting together a compelling manuscript.

Reviewer #3 (Remarks to the Author):

The authors have sufficiently addressed my suggestions. I have no major further comments. However, in Figs. 3 and 4 and Extended data Fig. 8, the p-values are written using a comma instead of a decimal point. Please correct this to the standard format using a decimal point.

We have modified the figures as requested. We thank the reviewer for her/his remarks and for helping us putting together a compelling manuscript.

Reviewer #4 (Remarks to the Author):

I co-reviewed this manuscript with one of the reviewers who provided the listed reports. This is part of the Nature Communications initiative to facilitate training in peer review and to provide appropriate recognition for Early Career Researchers who co-review manuscripts.

I appreciate the substantial effort made in revising the manuscript. The new experiments significantly strengthen the study, and my major previous concerns have largely been addressed.
In particular, the new in planta validation of the LCAT4–ATG8 interaction is an important addition. The co-IP data, the reduced interaction of the AIM mutant, and the failure of the AIM-mutated version to properly traffic/function provide a much stronger basis for the proposed role of LCAT4 in autophagic body processing.
The revised manuscript also provides much improved support for the vacuolar localization of LCAT3 and for the conclusion that its trafficking to the vacuole is largely independent of autophagosome formation. The added immunogold/TEM and *atg5* analyses are particularly helpful.
I therefore find the manuscript suitable for publication in principle. I only ask the authors to keep two points appropriately cautious in the final version:
1. The data are consistent with the idea that LCAT3 acts from the vacuolar side and that LCAT4 is delivered through autophagic structures, but the precise "outer leaflet / inner leaflet" assignment is still inferred rather than directly demonstrated. Statements on this point should therefore remain framed as a model or possibility.

We agree with the reviewer and modified the manuscript lines 438-440 from:
'Therefore, we propose that LCAT3 may recognize the outer leaflet of autophagic bodies while LCAT4 is found inside these compartments' to 'Therefore, we propose that LCAT3 may recognize the outer leaflet of autophagic bodies while LCAT4 is found inside these compartments, although this remains to be directly demonstrated'.

2. How LCAT3/LCAT4 selectively act on autophagic body membranes without affecting the tonoplast remains unresolved and should continue to be presented as an open mechanistic question.

We agree with the reviewer and modified our sentence line 497-500 according to her/his comment:

‘In addition, within the vacuolar lumen, whether and how a specific localization of LCAT3 and/or LCAT4 at the membrane of autophagic bodies could spatially determine the site of their activity and thereby prevent tonoplast hydrolysis remain open mechanistic questions to be investigated.’

We thank the reviewer for her/his remarks and for helping us putting together a compelling manuscript.
